# Design and modeling of a highly compact negative index floral shape metamaterial for flight navigation applications

**DOI:** 10.1038/s41598-025-88612-1

**Published:** 2025-02-06

**Authors:** M. Pallavi, Praveen Kumar, B. R. Shivakumar

**Affiliations:** 1 Department of Aeronautical and Automobile Engineering, Manipal Institute of Technology, Manipal Academy of Higher Education, Manipal, 576104 India; 2 Department of Electronics and Communication Engineering, Manipal Institute of Technology, Manipal Academy of Higher Education, Manipal, 576104 India; 3 Department of Electronics and Communication Engineering, NMAM Institute of Technology, Nitte (Deemed to be University), Nitte, 574110 India

**Keywords:** Antenna gain, Collision avoidance system, Flight navigation application, Metasurface, Metamaterial slab, Negative refractive index, Rectangular antenna, Traffic alert and collision avoidance system (TCAS), Electrical and electronic engineering, Electronics, photonics and device physics

## Abstract

The collision avoidance system (CAS) is a mandatory monitoring apparatus equipped in all aircraft to safeguard flight safety. The CAS scans the predefined regions in a systematic manner for a certain length of time to detect any approaching aircraft that could potentially pose a threat. Thus, CAS requires a focused multi-element radiator which can encompass the complete azimuth region. Recent years have seen a growing emphasis on enhancing the efficiency of CAS antennas because of several constraints, such as low gain (3.6 dB), larger dimensions, substantial side-lobe amplitude (− 7 dB), and challenges with beam adaptation. The current research strives to enhance the gain of a CAS antenna by incorporating the basic idea of metamaterials (MTMs). Therefore, a compact floral-shaped double negative (DNG) MTM design is proposed. The CAS antenna routes the signal throughout the complete azimuth region, so the designed MTM must be proficient to withstand its DNG characteristics for different incident angles. Hence, the proposed design is tested at various incident angles spanning between to along the azimuth region, at a deviation. The results indicate that the proposed structure retains its DNG behavior in the desired frequency range, regardless of the incident angles. The computed effective medium ratio of the structure is 13.47 at the CAS central frequency (1.06 GHz), highlighting its compactness and efficacy. Furthermore, to analyze the function of the structure on the antenna, the unit-element (UE) is expanded to a 5 × 4 array and deployed as an additional layer on the radiator at a predetermined distance. The addition of MTM to the radiator outperformed the conventional radiator by enhancing the antenna gain, by 2.6 dB, respectively. Additionally, to confirm the experimental findings, the UE and array designs are fabricated, and the fabrication results align closely with the simulation results.

## Introduction

Metamaterials (MTMs) are synthetic materials composed of subwavelength structures arranged in regular or irregular patterns, in contrary to organic substances that include atomic and molecular components. These arrangements possess remarkable potential to regulate electromagnetic (EM) waves based on particular standards such as perfect prism, selective absorption, magnetic susceptibility, electrical negativity, and refractive index (n) over the negative range, resulting in a variety of exceptional physical properties and useful gadgets^1–4^. The exceptional properties lead MTM as the best option for many fields including antenna performance improvements, SAR (Specific absorption rate) reduction, absorption, energy harvesting, sensing, super lenses, EM interface shielding, invisibility cloaking, RCS (radar cross section) reduction, satellite application, perfect imaging, optical vertex beam, polarization control^5–7^, etc.

The aforementioned attributes of MTM are mostly influenced by its innovative design principles, rather than the material’s composition. The examination of the changes in properties of EM waves when they interact with MTM structures can be carried out effectively by setting up suitable boundary conditions surrounding the MTM framework. Hence, while designing the MTM structure, it is feasible to modify the material properties (such as permittivity (ε), permeability (μ), and refractive index (n)) by tweaking the geometrical forms and periodic patterns of the MTM. Nevertheless, the exceptional characteristics of MTM give rise to new and unconventional electric (E) and magnetic (H) qualities that are unattainable in natural substances. These remarkable phenomena are accomplished by precisely structuring the split metallic circles in a spatial arrangement, with these loops functioning as miniature LC resonating circuits, resulting in the formation of resonant magnetic dipoles. Thus, the resulting MTM structure can be characterized as synthetic media with appropriate ' ε ' and ' μ ' values.

Theoretically, the material properties (ε and μ) of naturally occurring compounds are always positive; hence, they are classified as double positive compounds. In synthetic materials, the characteristics can be modified by altering the structural components; hence, in certain MTM designs, only one property may turn negative (termed single negative ‘SNG’), leading to either ‘ε’ negative (ENG) or ‘μ’ negative (MNG). In certain configurations, both ‘ε’ and ‘μ’ attain negative values, which results in double negative (DNG) MTM structures. Thus, unlike natural materials, synthetic materials have the ability to influence the EM field, resulting in discrepancies in EM wave propagation, particularly in subwavelength region.

A combination of square and concentric circle MTM is proposed in^8^ for the purpose of selectively blocking EM waves in the Wi-Fi spectrum. The structure is designed on 1.6 mm thick FR-4 base material with a total size of 20 mm × 20 mm (electrical dimension of 0.16λ × 0.16 λ) and an EMR (effective medium ratio) value of 6.25 (λ/6), making the structure relatively compact. An angular stability of 60% is recorded with a near-null cross-polarization (X-pol) effect in both TE (transverse electric) and TM (transverse magnetic) operating modes.

In^9^, a star-shaped MTM structure is proposed for the purpose of sensing solids as well as liquid substances. In this study, the structure is built on four different substrates: RO-6202, FR-4, RO-5880, and RO-3035, to better understand the effect of different dielectric constants and loss tangents on MTM performance, as well as how these materials will effectively distinguish between different liquids such as water, oil, milk, and energy drinks. As a result, the proposed MTM structure is applicable to a wide range of fields, including ecological surveillance, biomedical applications, the oil industry, and food nutrition management.

A fabric antenna coupled with a 2 × 2 AMC array was proposed for wearable electronics and health monitoring in^10^. The incorporation of artificial structures significantly enhanced radiator performance, including gain and overall efficiency, by regulating backward radiation. Moreover, the incorporation has markedly diminished the SAR (specific absorption rate) to ≤ 0.43 W/kg, thereby safeguarding human safety. In^11^, a compact SRR with a negative epsilon near null index (NI) is presented for multi-band applications. The structure is built on a 1.6 mm thick FR-4 substrate with a dimension of 10 mm × 10.5 mm (electrical dimension of 0.1λ × 0.1 λ), and an EMR value of 10.7 (~ λ/10). The proposed unit-element (UE) is expanded into a 4 × 4 array and utilized as a parasitic layer or superstrate to improve the gain and directivity of the radiator.

The spider web shape epsilon negative SRR is designed for multi-band microwave utilization in^12^. The proposed structure is built on 1.5 mm thick Rogers RO4350B dielectric material with a size of 10 mm × 10 mm (electrical dimension of 0.1 λ × 0.1 λ), which resulted in an EMR value of 10.7 (~ λ/11) making the structure extremely compact. Subsequently, the suggested UE is expanded into a 4 × 4 MTM array and positioned in close proximity to the radiator in order to enhance both the gain and BW of the antenna. The spiral-shaped MTM is presented in^13^ for airborne radar systems in order to boost overall antenna functionality. The construction is built on an RT/Duroid 5880 substrate with physical dimensions of 18.58 mm × 16.01 mm (electrical dimensions of 0.148 λ × 0.128 λ) and an EMR value of 6.75. The UE is expanded into a 6 × 6 array structure and placed on the radiator to enhance antenna characteristics such as impedance BW, gain, efficiency, and axial ratio BW.

In^14^, a near zero epsilon and negative index (NI) MTM design is presented for stealth and military applications to lower radar cross section (RCS) and enhance radiator overall gain and efficiency. The structure is built on a FR-4 substrate with an overall dimension of 6 mm × 6 mm (electrical dimensions of 0.138 λ × 0.138 λ) and an EMR value of 7.32. The use of a 5 × 5 MTM array as a superstrate on the radiator substantially improved antenna performance. A coupled holy cross and moon shape MTM structure is proposed in^15^ for wide range of applications, including stealth technology, satellite communication, defense, and aerospace. The design is built on FR-4 dielectric material with a physical dimension of 12.4 mm × 12.4 mm (electrical dimensions of 0.172 λ × 0.172 λ), and an EMR value of 5.8.

The negative epsilon and near-null index inverse G-shape MTM cell is presented for satellite and radar communication in^16^. The design is built on 1.6 mm thick FR-4 dielectric material with a physical dimension of 10 mm × 10 mm (electrical dimensions of 0.118 λ × 0.118 λ), and an EMR value of 8.47. MTM plays a pivotal role in improving the performance of antennas. Hence numerous distinct shapes of MTMs have been proposed, including chiral^17,18^, fractal^19^, monofilar—Archimedean^20^, circular SRR^21^, I-shape^22^, Circular ring^23^ and distinct MTM structures with switching topologies.

In the present work, a floral-shape negative index (or DNG) MTM structure is proposed for the Flight Navigation Application (FNA) system to enhance the overall performance of the radiator. The frequency range designated for flight collision avoidance system (FCAS) spans from 0.9 to 1.18 GHz, which is very near to the new satellite spectrum that resonates at 1.2 GHz. Hence, it is imperative to develop extremely precise MTM and radiator designs that operate within a designated frequency range and do not interfere with the GPS signal. The designed MTM depicts DNG behavior from 0.9 to 1.18 GHz, encasing only the spectrum essential for FCAS. The UE is extensively studied for the various incident degrees along the azimuth direction spanning from 0° to 360° with a step size 60°. The UE is subsequently expanded into 5 × 4 array, and all these structures are systematically analyzed to assess the coverage frequency and study their structural features. In order to verify the precision of the simulated findings, the MTM structure and array designs were constructed, and the results obtained align closely to the simulated values.

### Contribution

The significant aspects of the current work are emphasized below:

1. The negative index floral-shape MTM design operating at 1.06 GHz, with a frequency spectrum of 0.9–1.2 GHz is proposed for FNA.
2. The proposed MTM has a physical size of 21 × 21 × 1.6 mm^3^ and an EMR value of 13.47, making it a more compact structure than the conventional MTM design, which has a total size of 30 × 30 × 1.6 mm^3^^24^ and an EMR value of 9.4.
3. The proposed MTM is resilient to fluctuations in incident angles, which is critical for FNA.
4. The implementation of the proposed MTM array structure as an additional layer on the navigation antenna has increased the antenna gain by 2.6 dB while maintaining the reflection coefficient.
5. Furthermore, the present design can also be used to enhance the reflection coefficient, isolation of the MIMO topology, and to mitigate the impact of RCS (radar cross section) on RADOME encased antennas.

## Theoretical analysis and design procedure of MTM UE structure

This section contains a comprehensive analysis of the mathematical framework employed for estimating the theoretical size of the outermost ring, the final floral-shaped UE design, and the numerical modeling employed to study the dispersion/scattering parameters—which are of great importance in determining the material properties of the MTM. Furthermore, the study thoroughly examines the implications of distinctive strip lines on effective medium parameters (EMP) and examines the set of iterative phases that are required to construct the proposed structure.

### Conceptual framework for the floral-shaped MTM design

The proposed UE is a combination of four symmetrical floral-shaped closed stubs and L-shaped open stubs. Initially, the conventional numerical approach was utilized to determine the overall size of the outermost ring. Subsequently, the optimization technique was utilized for merging numerous structures with the outermost ring in order to attain the desired resonance frequency.

**Fig. 1:**
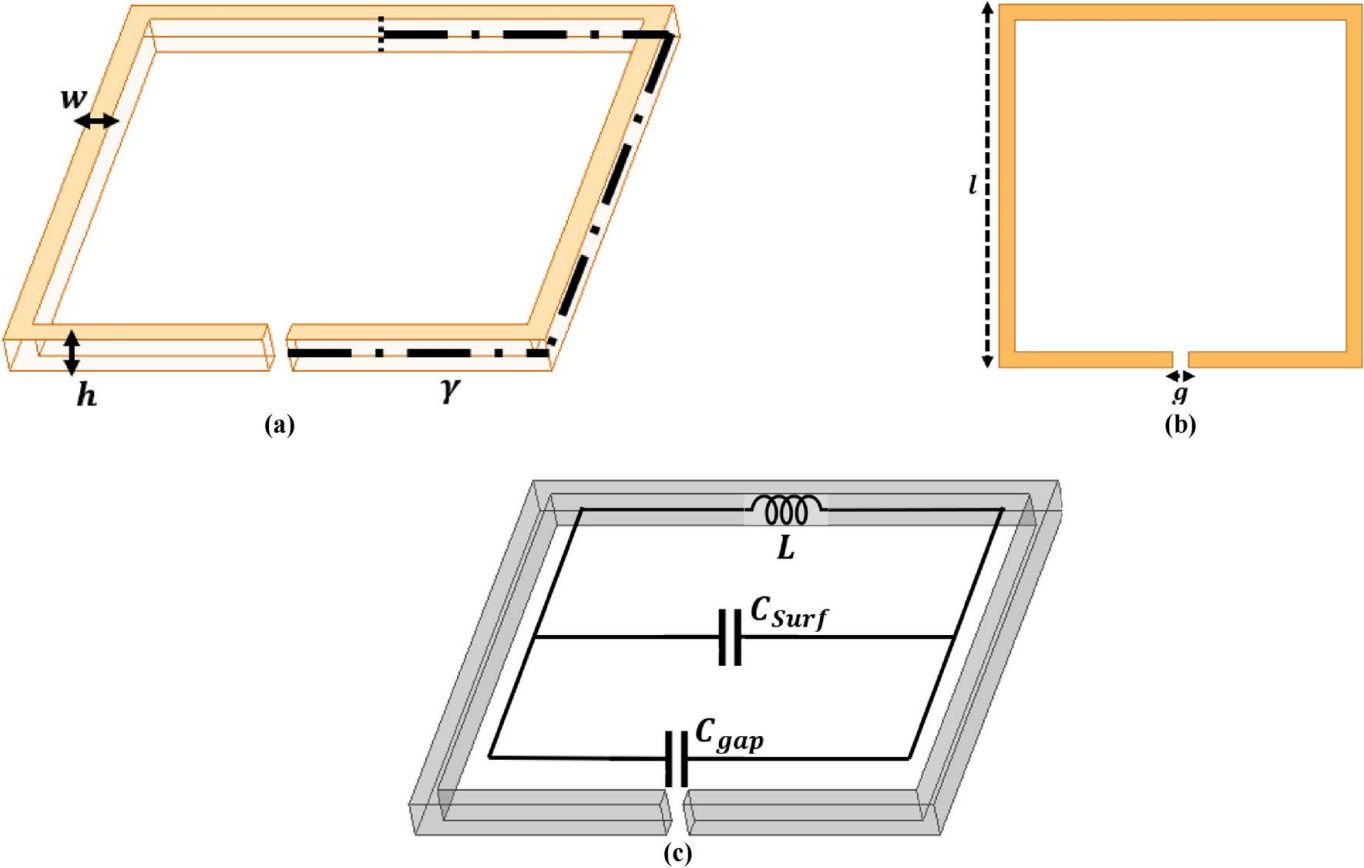
A 1 mm thickness single square split-ring for theoretical evaluation (**a**) Isometric-view, (**b**) Top-view, (**c**) Internal circuit of the structure.

The outermost ring or design in any MTM structure is crucial for determining the overall size, and the optimization technique is subsequently applied to generate a distinct structure with proper operating frequency. Hence, to analyze the effect of ring thickness ‘w’ and gap width ‘g’, a single square loop (SSL) with an area ‘a’ of 20 mm is taken into account. Figure 1a,b illustrates the isometric and top perspective of the SSL, whereas Fig. 1c shows the internal LC combination for the ring. At first, the magnetic field is applied to the SSL structure along the Y -axis to assess the distribution of EM field around the MTM structure. By appropriately integrating and tuning the values of LC, the EM field distribution can be altered, enabling the circuit to resonate at a specific frequency^25^.

1 $${\text{f}}_{0} = \frac{1}{{2\pi \sqrt {{\text{LC}}} }}$$

In Eq. (1), ‘L’ denotes inductance, which represents the overall length or area of the ring, and ‘C’ denotes capacitance,

which reflects the split in the ring surface. The inductance ‘L’ is expressed as

2 $${\text{L}} = {\upmu }_{0} {\text{a}}_{{\text{m}}} \left( {{\text{ln}}\frac{{8{\text{a}}_{{\text{m}}} }}{{{\text{h}} + {\text{w}}}} - 0.5} \right)$$

where ‘${\text{a}}_{\text{m}}$’ denotes the mean difference among two square loops, and it is given by

3 $${\text{a}}_{{\text{m}}} = {\text{a}} + {\text{w}}/2$$

where ‘$\text{a}$’ represents the overall area/length of the split ring, ‘$\text{w}$’ represents loop thickness, and ‘H’ represents substrate height.

The capacitance ‘C’ is the summation of gap and surface capacitances, and it is given by

4 $${\text{C}} = {\text{C}}_{{{\text{gap}}}} + {\text{C}}_{{{\text{surf}}}}$$

where

5 $${\text{C}}_{{{\text{gap}}}} = {\upvarepsilon }_{0} \left( {\frac{{{\text{wh}}}}{{\text{g}}} + \frac{{2\uppi {\text{h}}}}{{{\text{ln}}\left( {2.4{\text{h}}/{\text{w}}} \right)}}} \right)$$

6 $${\text{C}}_{{{\text{surf}}}} = \frac{{2{\upvarepsilon }_{0} {\text{h}}}}{{\uppi }}{\text{ln}}\frac{{4{\text{a}}}}{{\text{g}}}$$

where g denotes the split width.

By replacing μ_0_ (permeability of free space) with 4π × 10^–7^ N/A^2^, total area ‘a’ as 21 mm, mean area ‘a_m_’ as 21.5 mm, substrate height ‘h’ as 1.6 mm, loop width ‘w’ as 1 mm, loop gap ‘g’ as 0.5 mm, and ε_0_ (permittivity of free space) as 8.85 × 10^–12^ F/m, the theoretical value of ‘L’ and ‘C’ becomes 0.821 × 10^–9^ H and 1.041 × 10^–11^ F, respectively. Hence, the theoretical value of ‘f_0_’ in the absence of any coupling effect will be 1.722 GHz.

### Dynamic analysis of an MTM cell using dispersion relation

A dispersion diagram graphically illustrates the correlation between frequency ($\omega$) and wavevector ($k$) of EM waves travelling through a substance or a framework. In the frame of MTM, establishing a dynamic representation of the recurring structure is essential for examining wave propagation in the periodical lattice.

The subsequent derivation treats the proposed MTM cell as an assembly of rigidly interconnected Timoshenko beams. By adopting a conventional discretization method^26^, the kinetics expression of the MTM cell can be articulated as follows:

7 $$\left( {K - \omega^{2} M} \right)q = F$$

where $K$ denotes the global stiffness, $M$ denotes mass matrix, $\omega$ represents angular frequency, $q$ is the vector representation of node displacements, and $F$ is the vector representation force. For the MTM cell contained within a square lattice, as depicted in the figure, the displacement vector q can be expressed in the manner described below.

8 $$q = \left\{ {\begin{array}{*{20}l} {q_{l} } \hfill & {q_{r} } \hfill & {q_{b} } \hfill & {q_{t} } \hfill & {q_{i} } \hfill \\ \end{array} } \right\}^{T}$$

**Fig. 2:**
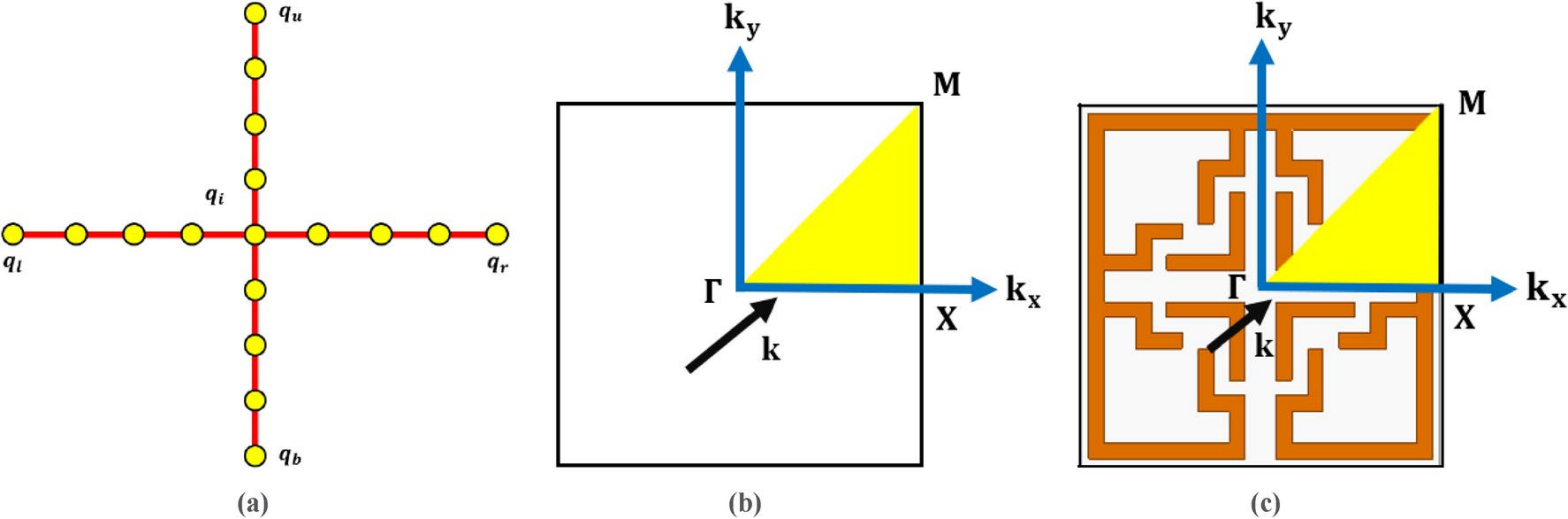
(**a**) Degree of freedom (DOF) of MTM cell, (**b**) Illustration of the 1st and Irreducible Brillouin Region (shaded yellow triangle), (**c)** Illustration of the Brillouin Region in the 2D cross section of the proposed MTM cell.

The subscripts $l, r, b, t,$ and $i$ represent the displacements associated with the left, right, bottom, top, and inner node of a MTM cell, respectively, as illustrated in Fig. 2a.

In accordance with Bloch’s theorem, periodic/recurring boundary conditions, that encompass the standardized displacements of the reference MTM cell and the equilibrium state of the standardized forces, can be expressed as:

9 $$q_{r} = e^{{k_{1} }} q_{1} \quad q_{t} = e^{{k_{2} }} q_{b}$$

Equation (9) affirms that, under periodic boundary conditions, the right end displacement of the MTM cell design can be determined by the displacement in the left end direction, and the top end displacement can be determined by the displacement in the bottom end direction. Equation (9) may be expressed in matrix notation as follows:

10 $$u = Aq_{r}$$

where $${q}_{r}$$ is $${\{{q}_{l} {q}_{b} {q}_{i}\}}^{T}.$$

Substituting Eq. (10) into Eq. (7) and presuming $F=0$ leads to the following result:

11 $$\left[ {K_{r} \left( {k_{1} , k_{2} } \right) - \omega^{2} M_{r} \left( {k_{1} , k_{2} } \right) q_{r} = 0} \right]$$

where ${K}_{r} \left({k}_{1}, {k}_{2}\right)$ denotes the minimized stiffness, and ${M}_{r}\left({k}_{1}, {k}_{2}\right)$ denotes the mass matrices. Equation (11) signifies an eigenvalue problem, the solution of which elucidates the dispersion characteristics related to the lattice. Generally, the wavenumbers ${k}_{1}$ and ${k}_{2}$ within the outline of the indeterminate section of the first Brillouin zone (Fig. 2b) are constrained in determining the frequency $\omega$.

Solving Eq. (9) yields the band structure for the specified ${k}_{1}$ and ${k}_{2}$, which can be employed to evaluate the dispersion characteristics. Furthermore, the group velocity delineates the directional characteristics of propagation of elastic wave and anisotropic features of the recurring structure. The directional properties of waves with elastic properties enhance the utility of periodic frameworks for vibration isolation. Therefore, it is imperative to examine the directional nature of wave propagation by evaluating the group velocity.

The group velocity delineates the wave direction properties of the lattice, and this can be articulated as follows:

12 $$c_{g} = \left( {\frac{\partial \omega }{{\partial k_{1} }} , \frac{\partial \omega }{{\partial k_{1} }}} \right)^{T}$$

**Fig. 3:**
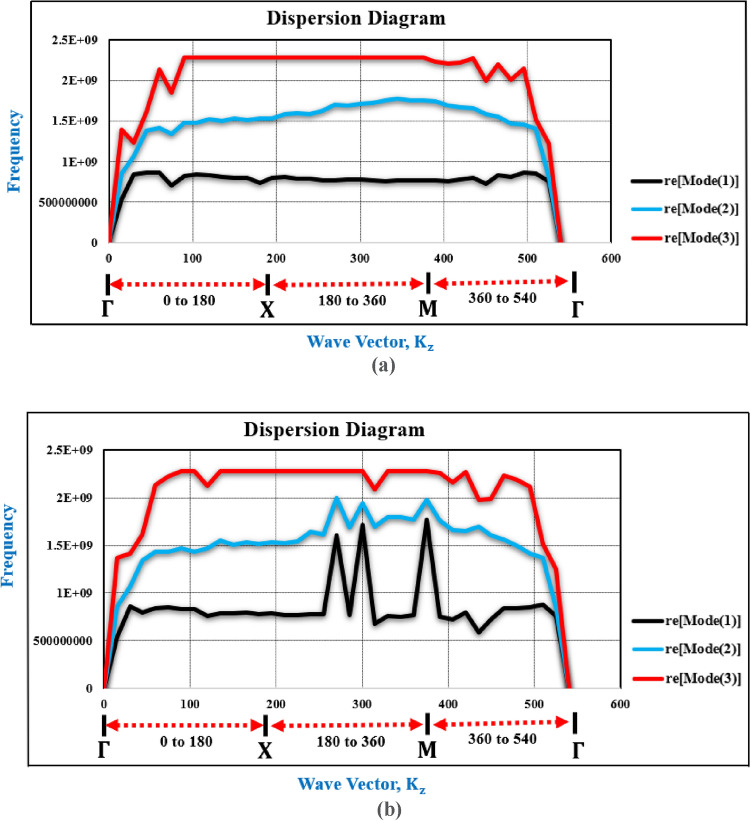
Dispersion Analysis of the MTM cell (**a**) Dispersion relation with three modes at 0.5 GHz (**b**) Dispersion relation with three modes at 1 GHz.

The dispersion characteristics are emphasized by the group velocity that are frequency-dependent, which delineates the anisotropy of the pertinent region in wave propagation. Figure 2c depicts the Brillouin Region within the two-dimensional cross-section of the proposed MTM cell. Figure 3 illustrates the corresponding dispersion relation (at the Brillouin Region) for three modes at two distinct frequencies one is at 0.5 GHz as illustrated in Fig. 3a and another is at 1 GHz as illustrated Fig. 3b.

### Design of floral-shaped UE

**Fig. 4:**
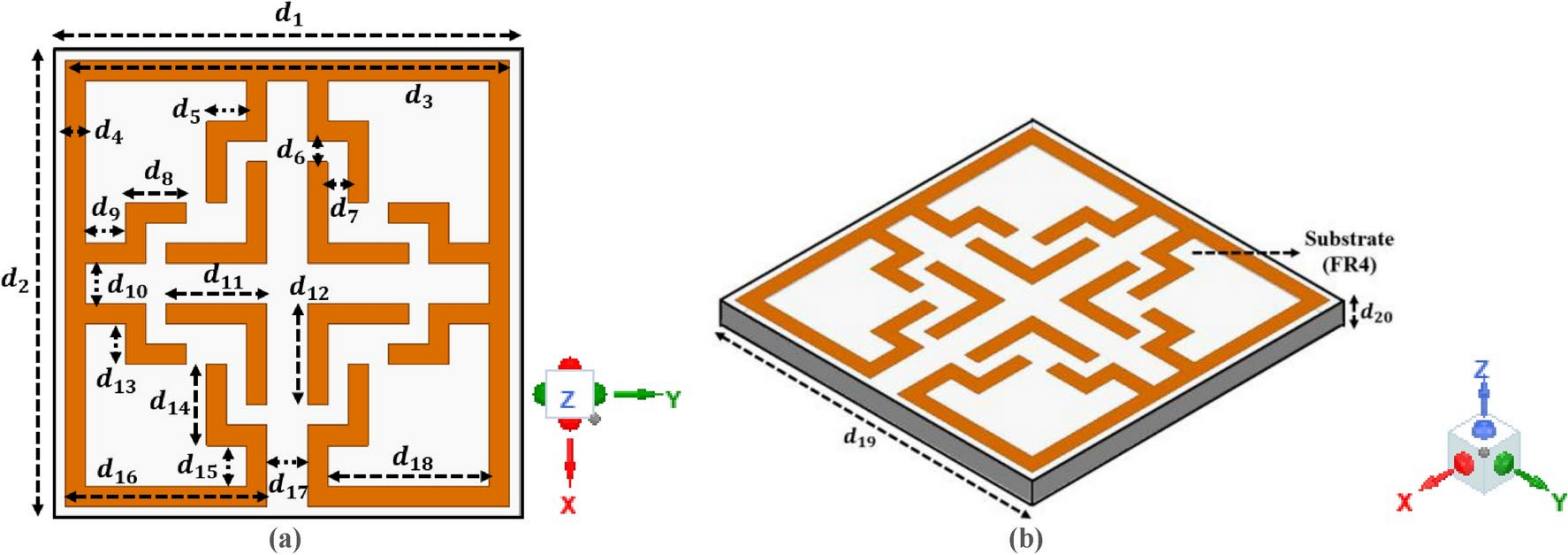
Proposed floral-shaped MTM Structure (**a**) Top-view (**b**) Isometric-view.

The proposed floral-shaped UE is depicted in Fig. 4 (Fig. 4a depicts top-view and Fig. 4b depicts isometric-view of the proposed structure). The UE and array structures are constructed using a 1.6 mm thick copper-clad FR-4 dielectric material, characterized by a dielectric constant (ε_r_) of 4.4 and a loss tangent (tan δ) of 0.032. The base material has a total dimension of 21 × 21 mm^2^, resulting in an electrical size of 0.08 × 0.08 λ^2^ at an operating frequency of 1.06 GHz. The UE is formed by combining four identical flower-shaped structures on four sides of the outer square loop with four identical L-shaped open stubs at the center of the loop. The total area of the outermost loop is 20 × 20 mm^2^ with 1 mm loop thickness.

The complete inductance is generated by the combination of floral-shaped metallic strips and L-shaped open stubs, while the single split and the gap between open stubs (L-shaped) and closed stubs (floral-shaped) contribute to the total capacitance value. The EM behavior of the presented UE is assessed utilizing the Ansys HFSS solvers, which rely on the FEM (finite element method) for computation. The structural specifications of both UE and array are presented in Table 1.

**Table 1:** Design specification of proposed structures.

DP	D	DP	D	DP	D	DP	D
d_1_	21 mm	d_12_	5 mm	d_23_	4.5 mm	d_34_	114 mm
d_2_	21 mm	d_13_	2 mm	d_24_	10 mm	d_35_	18.5 mm
d_3_	20 mm	d_14_	4 mm	d_25_	1 mm	d_36_	5 mm
d_4_	1 mm	d_15_	2 mm	d_26_	1 mm	d_37_	2 mm
d_5_	2 mm	d_16_	9 mm	d_27_	111 mm	d_38_	45 mm
d_6_	1 mm	d_17_	2 mm	d_28_	130 mm	d_39_	17 mm
d_7_	1 mm	d_18_	7 mm	d_29_	5 mm	d_40_	45 mm
d_8_	3 mm	d_19_	21 mm	d_30_	1 mm	d_41_	80 mm
d_9_	2 mm	d_20_	1.6 mm	d_31_	57.5 mm	d_42_	40 mm
d_10_	2 mm	d_21_	145 mm	d_32_	19 mm		
d_11_	5 mm	d_22_	94 mm	d_33_	91 mm		

DP: Design Parameters D: Dimensions.

**Fig. 5:**
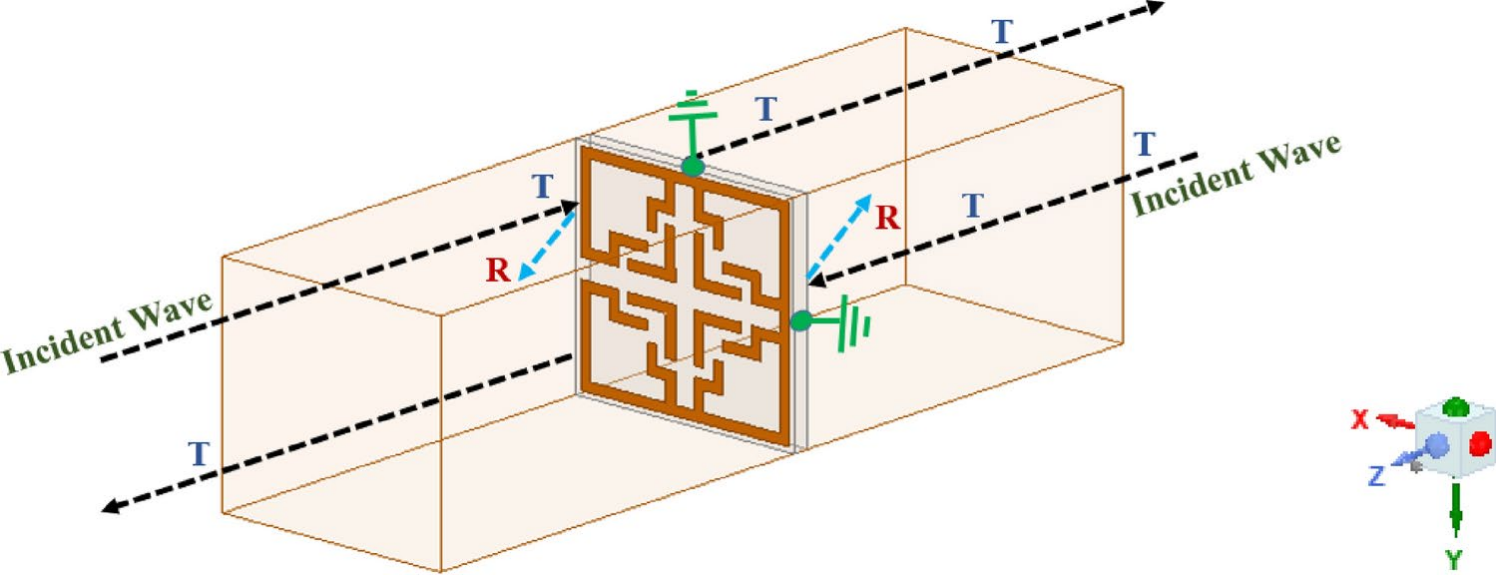
Simulation Setup for Extracting S -Parameters.

Figure 5 depicts the waveguide structure or framework used in this study for computing the scattering parameters (S-parameters) of the presented UE. The sides of the waveguide are subjected to two unique boundary circumstances: PEC (perfect electric conductor) and PMC (perfect magnetic conductor), in order to evaluate the MTM material properties and to attain periodicity. The PEC boundary constraints are applied on the sides of the waveguide that are parallel to the electric field, while PMC is applied on the faces that are parallel to the magnetic field. Finally, the MTM UE structure is positioned between either side (positive as well as negative) of the $\text{Z}$-axis, facilitating port excitation at both ends. All MTM designs (unit cell and array components) presented in this study will abide by similar boundary constraints.

The frequency band for the simulation is confined between 0.5 and 2 GHz based on the frequency spectrum allotted for the FNA application. The overall dimension of the proposed waveguide is 21 × 21 × 75 mm^3^ (0.08 × 0.08 × 0.26 λ^3^), and its height is confined to λ/13, which is pivotal for wave propagation.

### Methodology for EMP extraction

When EM waves strike the MTM slab, its constitutive parameters such as effective impedance (${\text{Z}}_{\text{eff}}$) and refractive index ($\text{n}$), can be determined by using Fresnel’s equations and inverting the S-matrix values^27^, as shown below.

13 $${\text{Z}}_{{{\text{eff}}}} = \pm \sqrt {\frac{{\left( {1 + {\text{S}}_{11} } \right)^{2} - {\text{S}}_{21}^{2} }}{{\left( {1 - {\text{S}}_{11} } \right)^{2} - {\text{S}}_{21}^{2} }}}$$

The polarity of the effective impedance '${\text{Z}}_{\text{eff}}$' is primarily determined by the behavior of the passive material, namely, the real part of effective ‘$\text{Z}$’ and the imaginary part of the effective ‘$\text{n}$’. To achieve a positive polarity, these components must be greater than zero.

The effective ‘$\text{n}$’ is derived from

14 $${\text{n}}_{{{\text{eff}}}} = \frac{1}{{{\text{k}}_{0} {\text{d}}_{{{\text{eff}}}} }}\left\{ {{\text{Im}}\left[ {\left( {{\text{e}}^{{{\text{in}}_{{{\text{eff}}}} {\text{k}}_{0} {\text{d}}_{{{\text{eff}}}} }} } \right)} \right] + 2{\text{m}}\uppi - {\text{iRe}}\left[ {\ln \left( {{\text{e}}^{{{\text{in}}_{{{\text{eff}}}} {\text{k}}_{0} {\text{d}}_{{{\text{eff}}}} }} } \right)} \right]} \right\}{\text{ m}} = 0,1, \ldots$$

where

15 $${\text{e}}^{{{\text{in}}_{{{\text{eff}}}} {\text{k}}_{0} {\text{d}}_{{{\text{eff}}}} }} = \frac{{{\text{S}}_{21} }}{{1 - {\text{S}}_{11} {\text{R}}_{01} }}{ },\quad {\text{R}}_{01} = ({\text{Z}}_{{{\text{eff}}}} - 1)/({\text{Z}}_{{{\text{eff}}}} + 1){ }$$

$\text{m}$ represents the complex branch index, ${\text{d}}_{\text{eff}}$ defines the MTM thickness, and ${\text{k}}_{0}$ is the wavenumber.

The imaginary component of ${\text{n}}_{\text{eff}}$ in Eq. (14) is unaffected by the division of the exponential value, allowing for precise estimation. The rear portion, in contrast, yields varied responses owing to branch ambiguities. Subsequently, the Kramers–Kronig method^28^ is adopted to evaluate these responses and ascertain the distinctiveness of the significant parameters. In the end, the validated ${\text{n}}_{\text{eff}}$ and ${\text{Z}}_{\text{eff}}$ findings are used to calculate effective ‘ε’ and ‘μ’ using the equations below.

After evaluating ${\text{z}}_{\text{eff}}$ and ${\text{n}}_{\text{eff}}$, the findings will be used to compute the effective material properties such as ${\upvarepsilon }_{\text{eff}}$ and ${\upmu }_{\text{eff}}$ using the following expressions:

16 $$\upvarepsilon _{{{\text{eff}}}} = \frac{{{\text{n}}_{{{\text{eff}}}} }}{{{\text{z}}_{{{\text{eff}}}} }}$$

17 $$\upmu _{{{\text{eff}}}} = {\text{n}}_{{{\text{eff}}}} .{\text{z}}_{{{\text{eff}}}}$$

### Design technique for the floral-shaped MTM

**Fig. 6:**
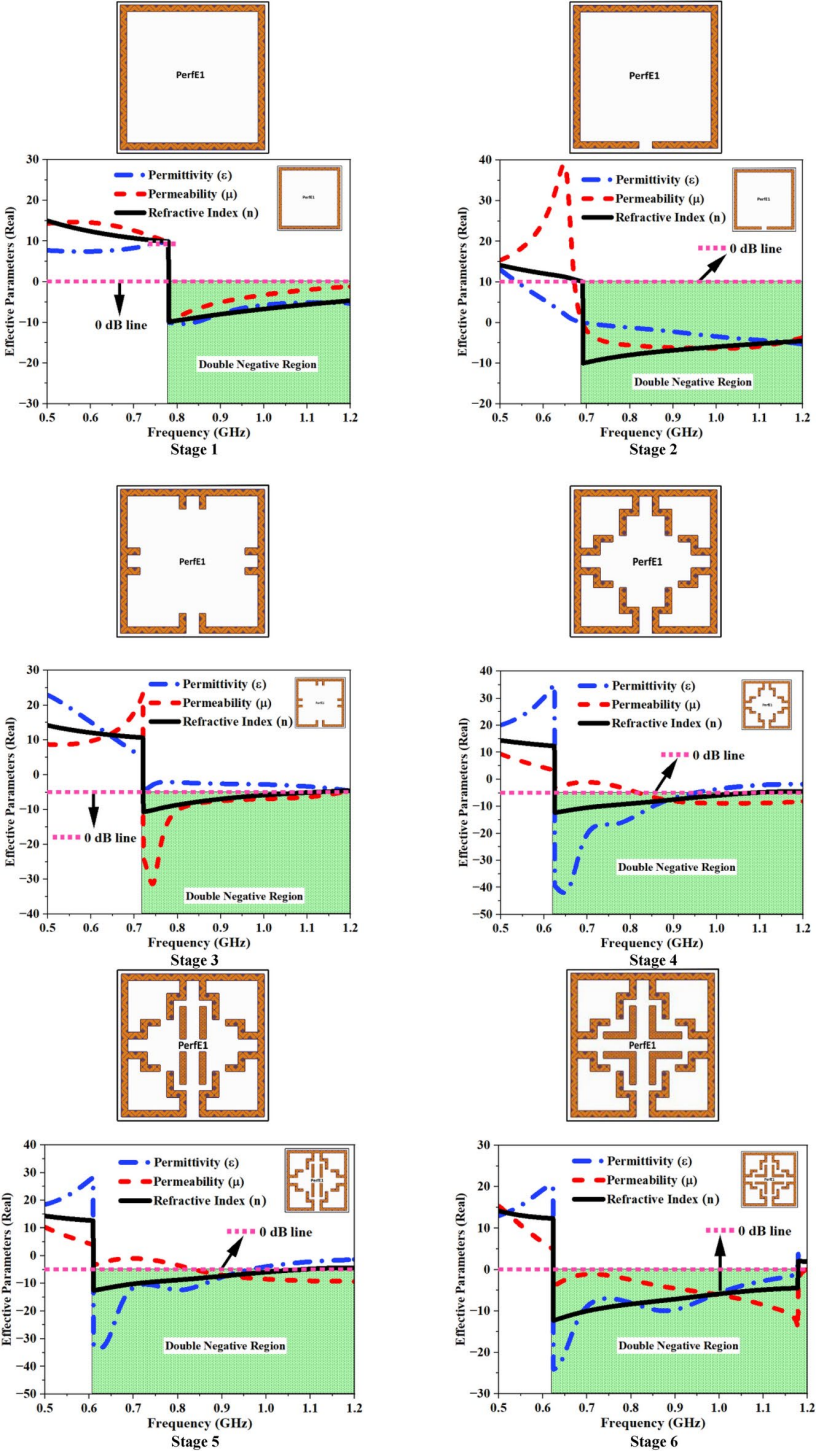
Development stages of MTM UE with its corresponding EMPs.

In the present research, an iterative method was utilized to assess several MTM cell combinations in order to determine the optimal MTM structure. The primary design criteria for this study include achieving a precise frequency band that covers the complete range permissible for FNA usages, appropriate S-parameter results, DNG material quality, and a compact design with a significant EMR value. To comply the MTM construction with these prerequisites, the design process underwent multiple structural alterations. These adjustments dealt with controlling the length and width of each single strip line, split gaps, and the implications of mutual coupling, as shown in Fig. 6.

Initially, a single closed square loop was developed using theoretical assessment as given in Stage 1, and the structure resonated at 1.947 GHz with a reflection coefficient (S_21_) of − 42.89 dB, displaying DNG properties between 0.78 and 1.72 GHz. A single split is etched in Stage 2 to move the resonating frequency towards the FNA central frequency (1.06 GHz), and the inclusion of this has driven the resonance to 1.19 GHz with − 37.46 dB S_21_ value, displaying DNG behavior between 0.69 and 1.34 GHz.

In Stage 3, two identical stubs are placed on four sides of the split ring to lower the resonant frequency by increasing the inductance. The insertion of these stubs lowered the resonant frequency to 1.16 GHz, with an S_21_ magnitude of − 35.94 dB. The identical stubs on the four sides of the square ring have been extended to form a floral-shaped structure, as shown in Stage 4, to increase the total inductance of the structure. These extended stub lengths have further reduced the resonating frequency to 1.09 GHz with a − 34.59 dB S_21_ value, and the DNG behavior for this structure is limited to 0.63–1.22 GHz. In addition, four similar open stubs were added to the middle of the floral-shaped structure, as shown in Stage 5, to allow for slight deviation and fine tuning of the resonant frequency. The introduction of these open stubs reduced the resonant frequency to 1.07 GHz, with a − 34.76 dB S_21_ value and a DNG spectrum that varied from 0.61 to 1.21 GHz.

**Fig. 7:**
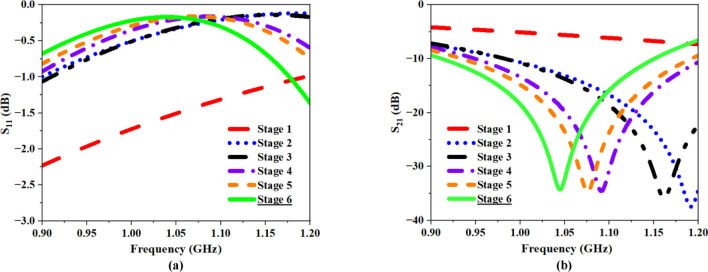
S-Parameter plots at various MTM development stages (**a**) Transmission Coefficient, (**b**) Reflection Coefficient.

In Stage 6, the open stubs in the center are extended as L-shaped stubs, and the increased length of these stubs has reduced the resonating frequency to 1.048 GHz with − 34.29 dB S_21_ value, demonstrating DNG behavior between 0.62 and 1.18 GHz. The present setup encompasses a frequency range of 0.91 and 1.15 GHz, making it well-suited for the FNA application. The corresponding S-parameters for all six stages are illustrated in Fig. 7a,b.

The FNA application necessitates a radiator that is capable of directing radiation in all directions around the aircraft to monitor the entire surrounding area. Therefore, proposed MTM configuration is assessed using various incident angles, ranging from 0 to 360° degrees, with a 60° step increment.

**Fig. 8:**
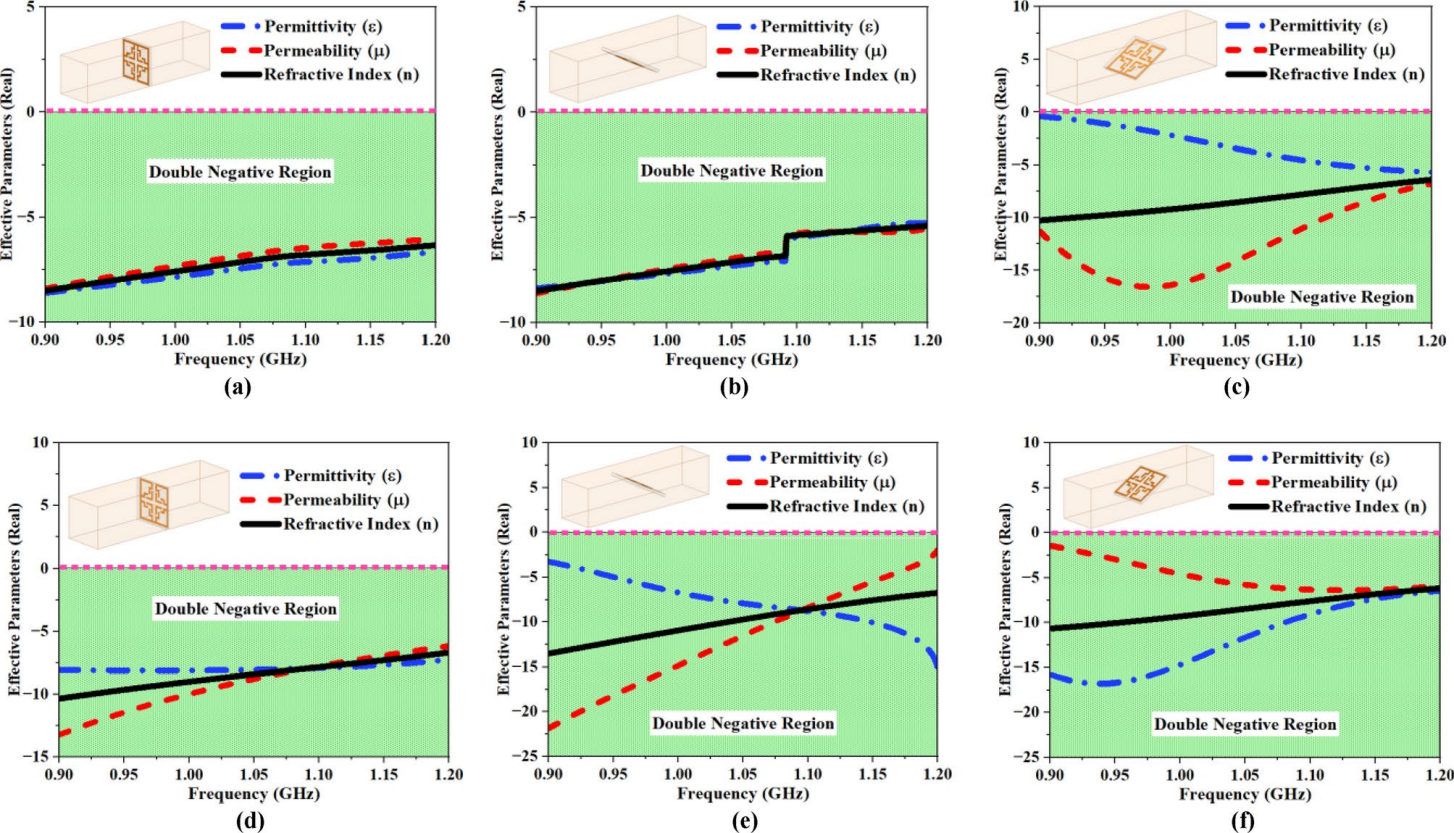
MTM UE analysis at various incident angles (IAs) and its impact on EMP (**a**) or (**b**) (c) (**d**) (**e**) & (**f**).

The EMP for the proposed MTM cell at different incident angles (0°, 60°, 120°, 180°, 240°, and 300°) is illustrated in Fig. 8a–f, and for each incident angle, the proposed structure exhibits the behavior of a DNG material within the frequency range of 0.5–1.5 GHz, which covers the entire spectrum of FNA. Table 2 presents a summary of the material properties (‘ε’, ‘μ’, and ‘$\text{n}$’) recorded at various incident angles.

**Table 2:** MTM material properties recorded at different incident angles.

Angle (φ)	Range of Negative ε (in GHz)	Range of Negative μ (in GHz)	Range of Negative n (in GHz)	Amplitude @ 1.06 GHz			BW of DNG region (GHz)
				ε	μ	n	
0° or 360°	0.72–1.31	0.69–1.38	0.69–1.33	− 7.4	− 6.8	− 7.1	0.64
60°	0.78–1.36	0.63–1.34	0.64–1.35	− 7.2	− 6.9	− 7.1	0.71
120°	0.83–1.28	0.76–1.29	0.76–1.29	− 3.7	− 13.6	− 8.4	0.53
180°	0.69–1.33	0.64–1.35	0.65–1.33	− 2.6	− 16.4	− 9.2	0.68
240°	0.81–1.39	0.63–1.23	0.72–1.33	− 8.1	− 8.6	− 8.3	0.61
300°	0.69–1.26	0.84–1.31	0.69–1.31	− 11.1	− 5.9	− 8.3	0.69

## Results and discussion

This section discusses the analogy between the simulated and measured outcomes of both the UE and array structures of the presented MTM. The subsequent paragraph offers an elaborate elucidation of crucial topics, such as the impact of separation distance (both horizontally and vertically) between UE on the antenna performance of the array structure, as well as a comprehensive description of the effect of mutual coupling. In addition, the influence of different structural factors, such as split width, width, length, and shape of strip lines, as well as the impact of various dielectric constants on material properties, is comprehensively addressed.

### Floral-shaped UE structure analysis

**Fig. 9:**
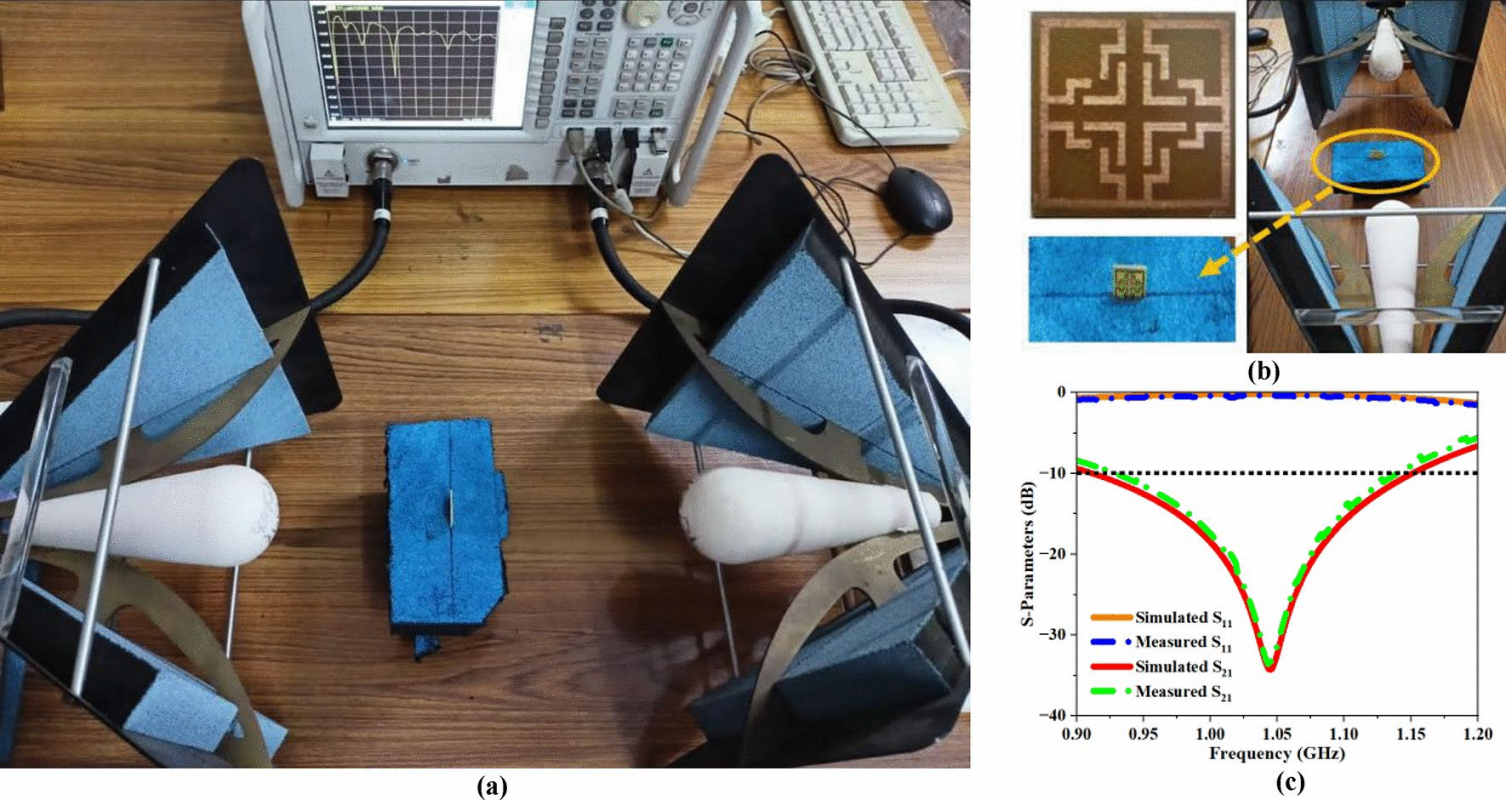
(**a**) Measurement setup (**b**) Fabricated UE structure (**c**) Simulated and measured S -parameter plot.

Figure 9a,b illustrate the developed UE framework, the experimental arrangement for data collection, and the relationship between the simulated and measured outcomes. The measured and simulated outcome demonstrates a significant level of concurrence, as shown in Fig. 9c. Furthermore, the EMP of the MTM is assessed by examining the variation in S-parameters resulting from the interaction between the propagating EM signal and the UE structure. A mathematical model is utilized to develop a computer program that derives ${\upvarepsilon }_{\text{eff}}$, ${\upmu }_{\text{eff}}$, ${\text{n}}_{\text{eff}}$, and ${\text{z}}_{\text{eff}}$ based on recorded S-parameter values.

**Fig. 10:**
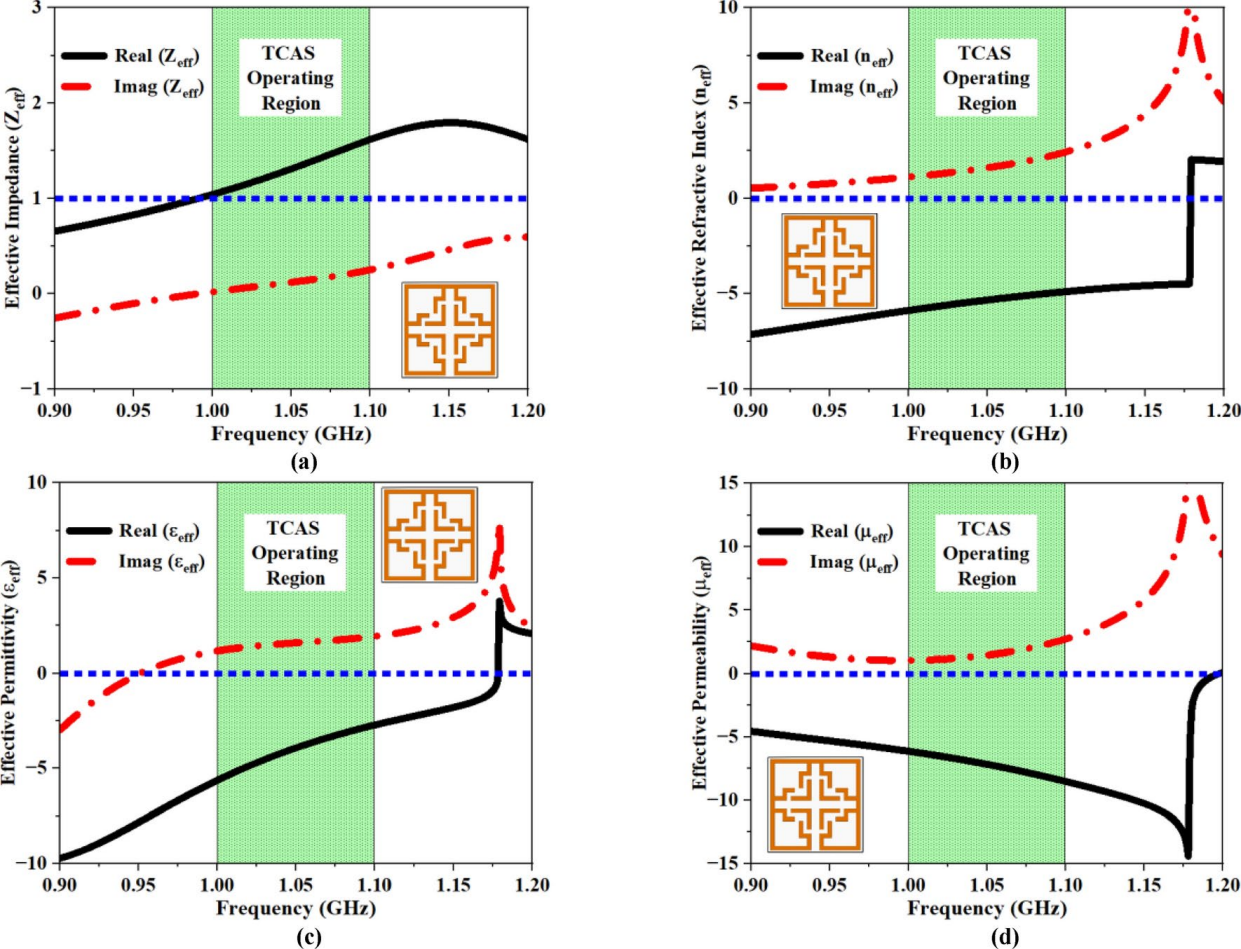
Simulated real and imaginary EMP values for UE (**a**) Z_eff_, (**b**) n_eff_, (**c**) ε_eff,_ (**d**) μ_eff_.

Figure 10 depicts the real and imaginary components of all four EMPs. Figure 10a provides the effective impedance variation across the FNA spectrum. The plot illustrates that the real part of the ‘$\text{Z}$’ is positive within the frequency range of 1–1.1 GHz, with a magnitude ranging from $1.04$ to $1.62$. Meanwhile, the imaginary part of the impedance remains close to zero, with a value ranging from $0.02$ to $0.24$. The magnitude of the absolute ${\text{Z}}_{\text{eff}}$ recordings at the operating frequency (1.06 GHz) is 1.37, while the imaginary part of ${\text{Z}}_{\text{eff}}$ is $0.14$. In conceptual terms, the minimum ${\text{Z}}_{\text{eff}}$ at the relevant spectrum demonstrates the passive characteristic of the MTM across the spectrum. As shown in Fig. 10b, the absolute value of the effective index (${\text{n}}_{\text{eff}}$) is negative between 1 and 1.1 GHz, with an amplitude ranging from − 5.88 to − 4.89, whereas the imaginary n_eff_ value is positive across the same spectrum, with an amplitude ranging from 1.12 to 2.44. The absolute and imaginary n_eff_ values at 1.06 GHz are − 5.23 and 1.73, respectively. The deviation of effective permittivity (${\upvarepsilon }_{\text{eff}}$) over the frequency spectrum is shown in Fig. 10c. Here, the absolute ${\upvarepsilon }_{\text{eff}}$ data is negative between 1 and 1.1 GHz, with an amplitude ranging from − 5.63 to 2.72, while the imaginary ${\upvarepsilon }_{\text{eff}}$ value is positive across the same spectrum, with an amplitude ranging from $1.17$ to $1.93$. The absolute and imaginary ${\upvarepsilon }_{\text{eff}}$ values at 1.06 GHz are − 3.65 and 1.64, respectively. Figure 10d illustrates the variance of effective permeability (${\upmu }_{\text{eff}}$). The absolute ${\upmu }_{\text{eff}}$ value is negative between 1 and 1.1 GHz, with a magnitude variation of − 6.13 to − 8.52, while the imaginary ${\upmu }_{\text{eff}}$ value is positive, with a magnitude range of 1.05 to 2.72. At 1.06 GHz, the imaginary and absolute ${\upmu }_{\text{eff}}$ values are 1.63 and − 7.41. Therefore, the proposed UE complied with all the essential prerequisites to function as an MTM, which include positive ${\text{Z}}_{\text{eff}}$, negative ${\upvarepsilon }_{\text{eff}}$, negative ${\upmu }_{\text{eff}}$, and negative ${\text{n}}_{\text{eff}}$, respectively.

**Fig. 11:**
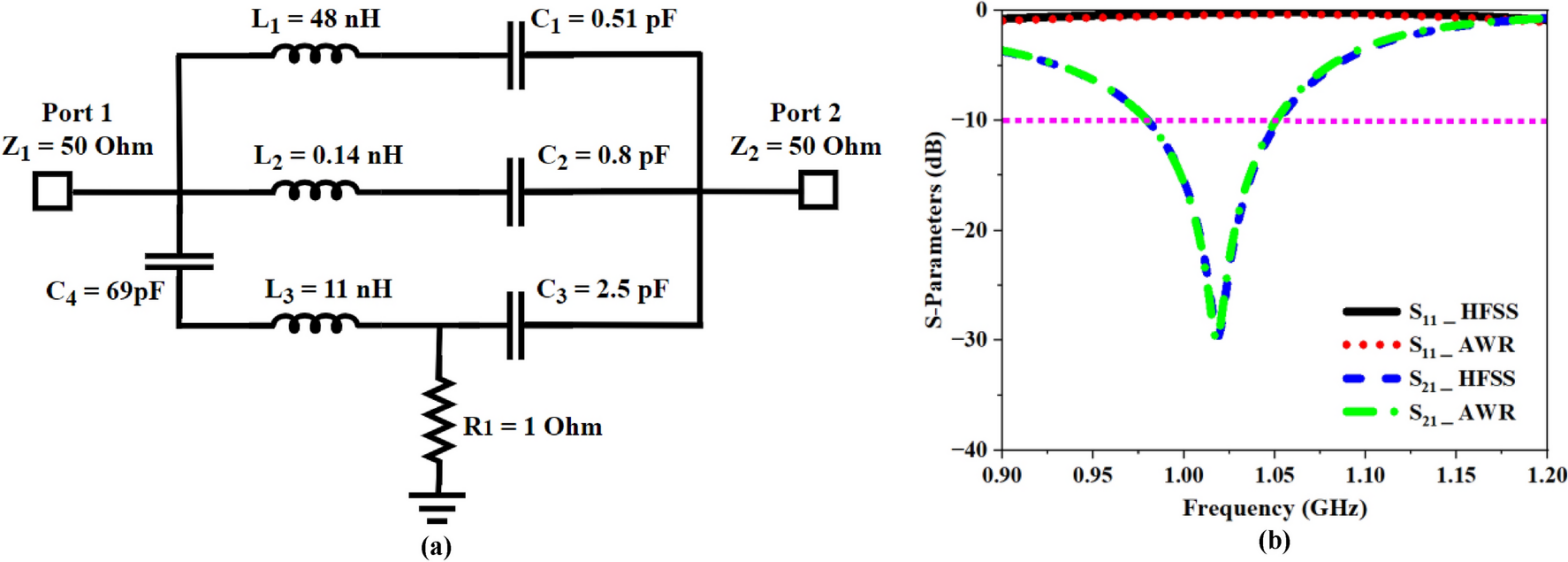
Circuit Prototype (**a**) Schematic of the proposed MTM cell equivalent circuit, (**b**) Recorded AWR and HFSS S-Parameter plots.

Figure 11a depicts the internal LC resonant circuit for the proposed MTM cell. The S-parameter plot from the LC circuit is compared to the recorded simulator (HFSS) S-parameter values, as seen in Fig. 11b, and both graphs align perfectly.

When designing an MTM structure, it is imperative to consider the EMR value as a pivotal aspect. This value signifies the compactness and structural efficiency of structure, and in order to achieve MTM qualities, the EMR value must be larger than 4. The FNA functions at a central frequency of 1.06 GHz, allowing the UE length to reach a maximum of 74 mm, based on the theoretical notion (EMR = 4). Therefore, by decreasing the total length of the UE, the EMR value can be significantly enhanced. The subsequent equation is utilized to evaluate the EMR value:

18 $${\text{EMR}} = \frac{{{\text{Wavelength}} \left(\uplambda \right)}}{{{\text{Unit}} - {\text{cell}}\,{\text{length}} \left( {\text{L}} \right)}}$$

The current study achieved the required MTM feature at 1.06 GHz while keeping the overall size of the UE at 21 mm. This size corresponds to a maximum EMR measurement of 13.47, confirming the compactness and structural efficiency of the present design. Furthermore, high EMR offers various advantages, including increased characteristic homogeneity, less spatial dispersion on entire MTM efficiency, a moderate electrical dimensionality that mitigates production constraints, and less coupling influence owing to scaling down.

### 5 × 4 MTM Array structure analysis

**Fig. 12:**
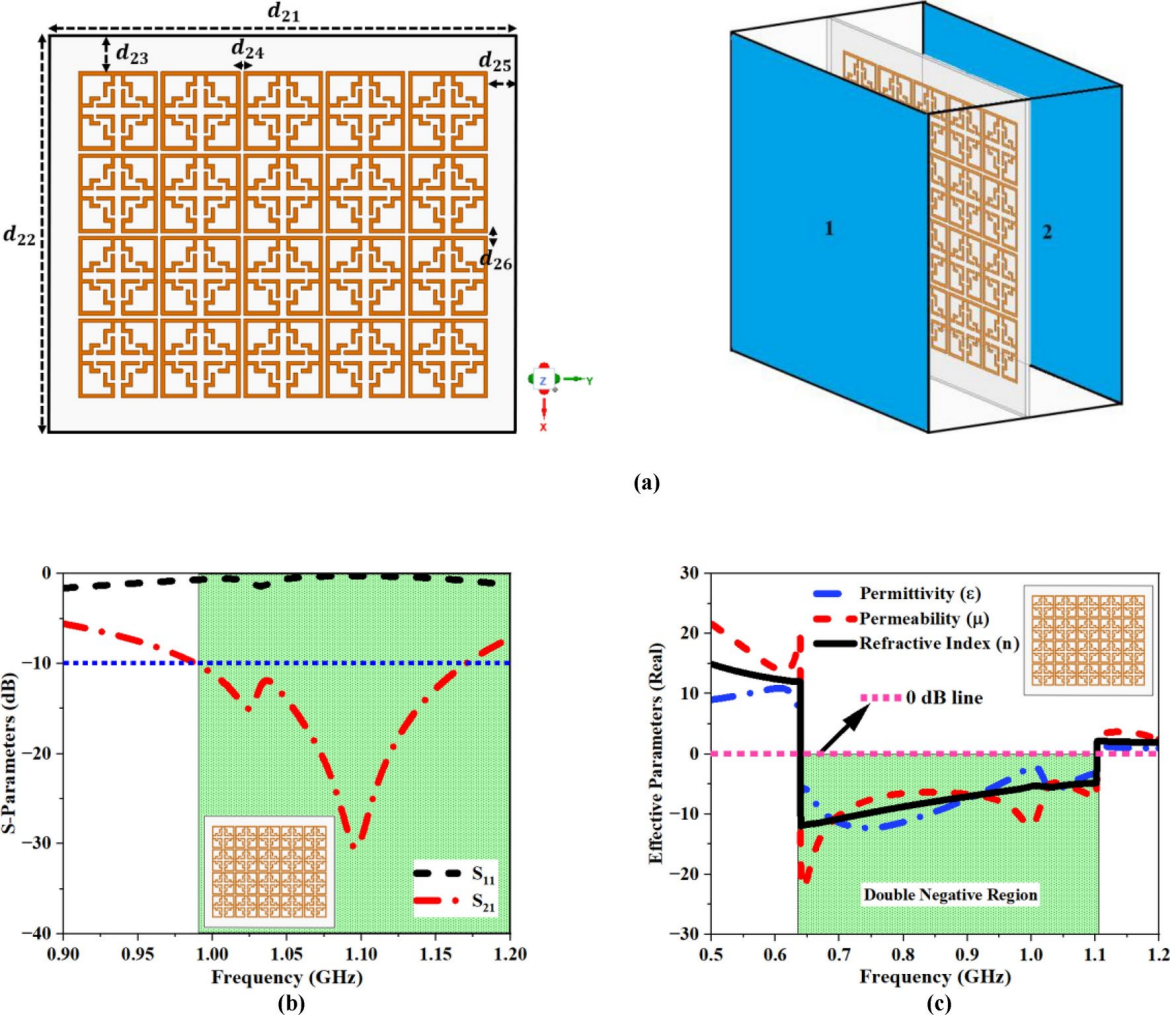
MTM array analysis (**a**) 5 × 4 array structure and measurement setup, (**b**) Simulated S -Parameter result. (**c**) Real EMP plot for 5 × 4 array.

The first plot of Fig. 12a depicts a simulated $5\times 4$ array topology for a floral-shaped MTM. Each UE is separated by 1 mm between adjacent cells, both horizontally and vertically, and the MTM slab has total dimensions of $130\times 111\times 1.6\,{\text{mm}}^{3} (0.46\times 0.39\times 0.011{\uplambda }^{3})$.

**Fig. 13:**
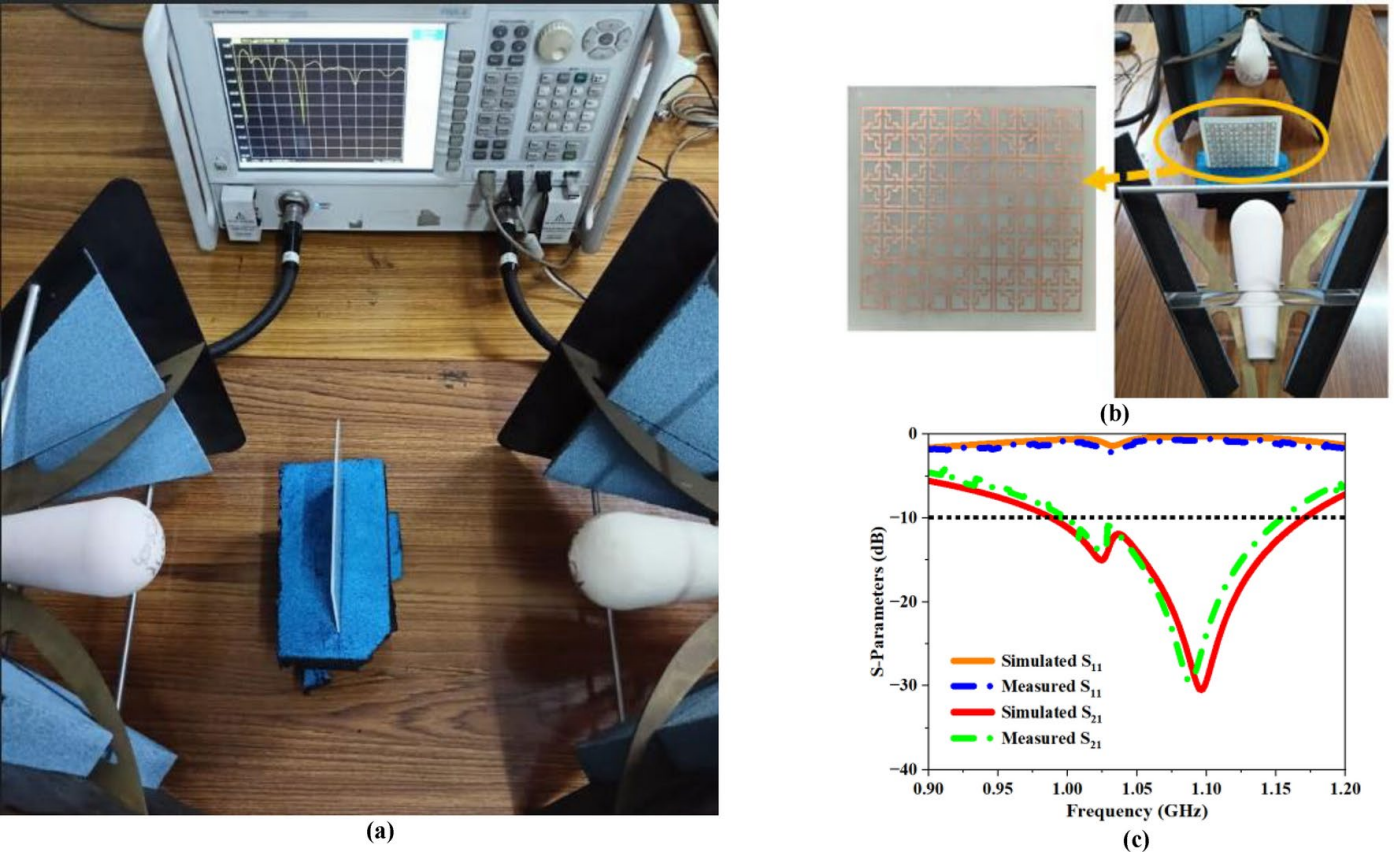
(**a**) Measurement setup (**b**) Fabricated 5 × 4 array structure (**c**) Simulated and Measured S -Parameter plot.

The second plot depicts the simulation setup utilized for analyzing and evaluating the outcome of the array design. The S-parameter plot for the array design is depicted in Fig. 12b. Based on the recorded findings, the structure resonates at 1.09 GHz with an amplitude peak of $-30.52\,\text{dB}$. The recorded results are subsequently used to construct the EMP plot depicted in Fig. 12c. The real value of ‘ε’ remains negative within the frequency range of 0.64 GHz to 1.11 GHz. At the resonance frequency, the computed magnitude for ‘ε’ is $-3.52$. The real value of ‘μ’ turns negative at a frequency of 0.64 GHz and remains negative until 1.11 GHz. The estimated magnitude of ‘μ’ at the resonance frequency is − 6.76. In addition, the real value of ‘n’ turns negative at a frequency of 0.64 GHz, the negative phase persists till 1.11 GHz, yielding a peak magnitude of − 4.88 at the resonance frequency. The simulated findings clearly demonstrate that the 5 × 4 array structure displays Left-Handed Material (LHM) behavior across the frequency range of 0.64–1.11 GHz. Figure 13a,b shows the fabricated 5 × 4 MTM array prototype, the apparatus used for measurements, and the simulated and measured S -parameter findings. As depicted in Fig. 13c, the measured findings exhibited a significant correlation with the simulated outcomes.

### Study of mutual coupling

**Fig. 14:**
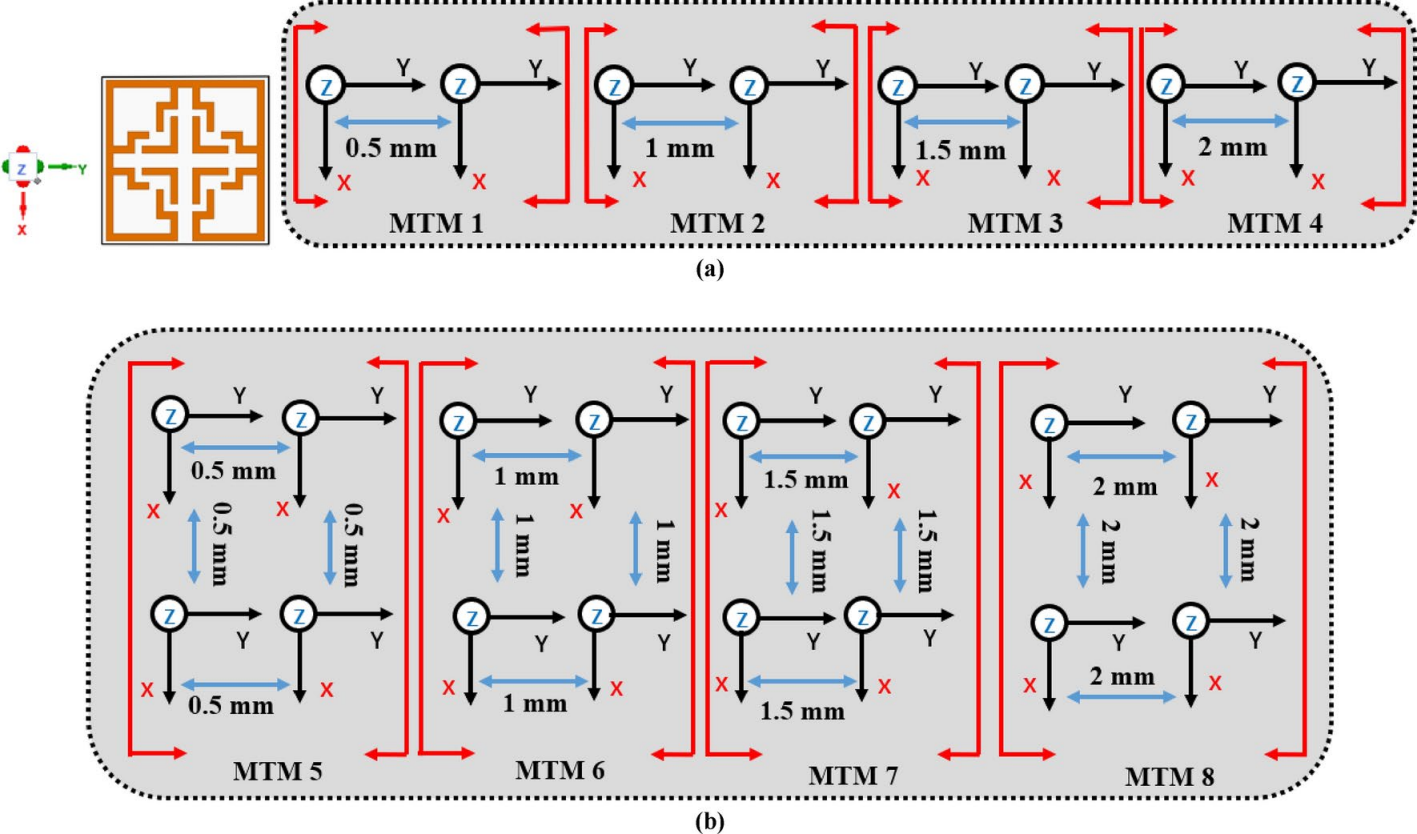
Analysis of coupling effect between UEs (**a**) 1 × 2 array (**b**) 2 × 2 array.

This section comprehensively describes the influence of coupling among inter-MTM UEs across various array configurations. At first, four identical $1\times 2$ array architectures were considered, each with distinct coupling distances as illustrated in Fig. 14a.

**Fig. 15:**
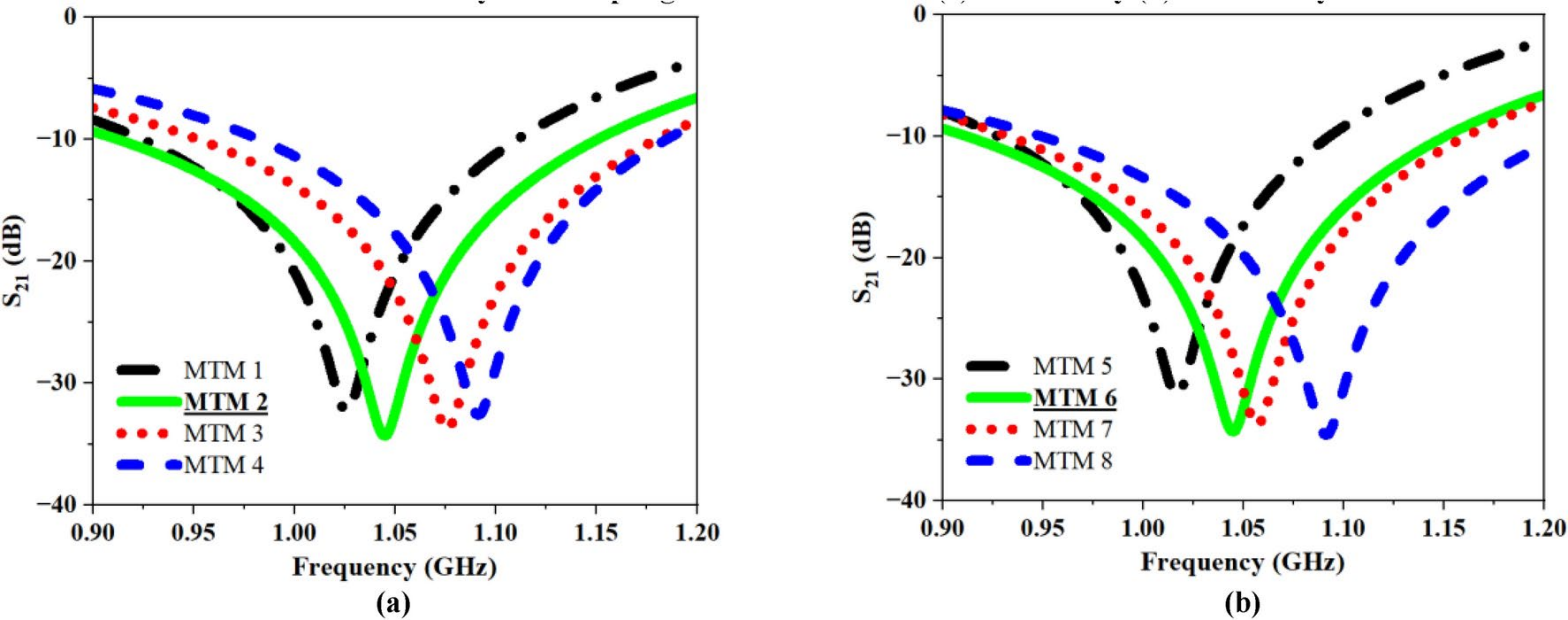
Recorded S_21_ results for different coupling distance (**a**) 1 × 2 array structure (**b**) 2 × 2 array structure.

Figure 15a shows the computed ${\text{S}}_{21}$ graph for this case, highlighting how variations in coupling distance affect the S-parameter. The separation between two UEs is horizontally altered from 0.5 to 2 mm. The resonant frequencies obtained at varied spacings are 1.02 GHz, 1.05 GHz, 1.07 GHz, and 1.09 GHz, with S_21_ peak amplitudes of − 32.33 dB, − 34.92 dB, − 34.85 dB, and − 33.91 dB, respectively. Upon data analysis, the horizontal spacing between two UEs is fixed to 1 mm in our research. Subsequently, to ascertain the precise horizontal and vertical spacing between each UE, an iterative approach was carried out, considering the 2 × 2 array structure (Fig. 14b). Figure 15b displays the computed S_21_ plot for the given scenario, demonstrating the impact of varying coupling distance on the S -parameter. The gap between two UEs is changed from 0.5 to 2 mm on both the horizontal and vertical axes. The resonant frequencies obtained at varied spacings are 1.01 GHz, 1.05 GHz, 1.06 GHz, and 1.09 GHz, with S_21_ peak amplitudes of − 31.13 dB, − 34.91 dB, − 34.87 dB, and − 36.12 dB, respectively. After reviewing the data, the horizontal and vertical spacing between two UEs are set to 1 mm.

### Analysis of various parameters and their effect on operating frequency

In this section, a comprehensive parametric analysis was carried out to examine the significance of various structural parameters on the resonant frequency and to gain a thorough grasp of the fundamental concepts involved in the construction of MTM structures. The present study employed a parametric optimization technique to regulate the overall dimensions of the proposed structure. The design has undergone multiple simulations, assessing various parameters including the impact of multiple gap size and their nonexistence on the operating frequency, the incorporation of multiple closed and open stubs in the framework, and the influence of different dielectric coefficients on material characteristics.

#### Analysis of capacitive gaps

Prior to finalizing the proposed structure, the design underwent multiple optimization stages to develop a distinctive and compact structure. A study was initially conducted to ascertain the effect of capacitance gaps (CGs) at various places on performance.

**Fig. 16:**
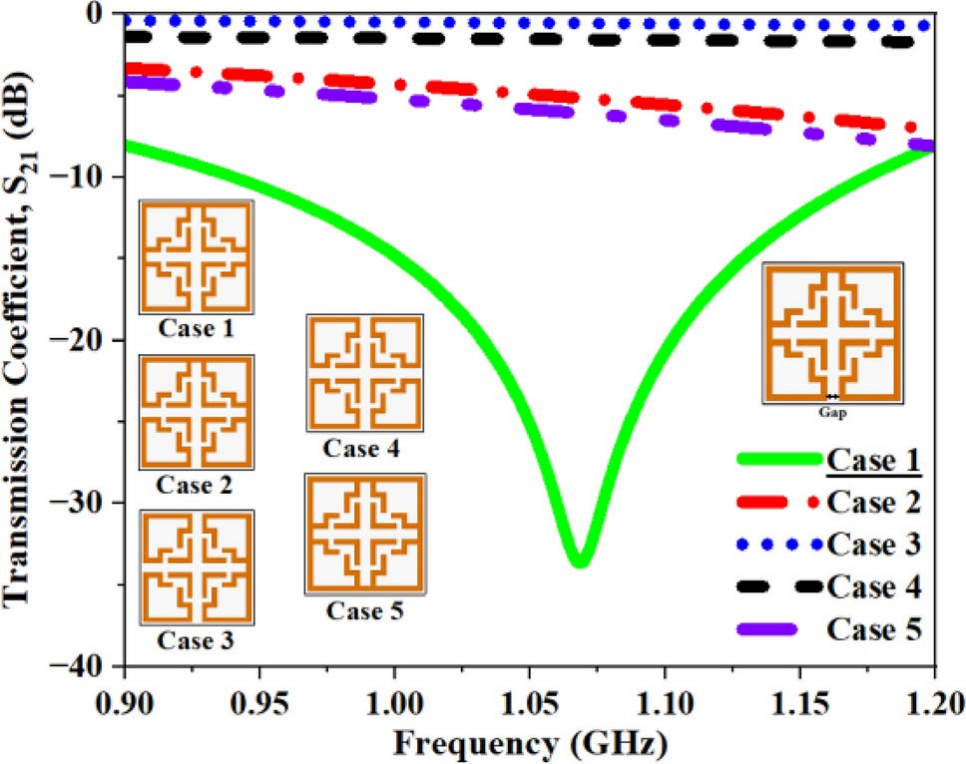
Computed results of S_21_ and f_0_ for multiple capacitive gaps.

At first, a single 2 mm long split is etched at the bottom side of the outermost ring. Later, the same dimension split is etched on four edges of the square structure sequentially to assess the effect of multiple splits, and in final case, the split is sealed on all sides to assess its significance in the proposed structure. Figure 16 shows the corresponding S_21_ plots for multiple cases and the computed results for different cases are summarized in Table 3. In case 1 (one split at the bottom edge), the structure resonates at 1.07 GHz with a maximum amplitude of − 33.65 dB. In cases 2, 3, and 4, the inclusion of extra splits along each side of the square has shifted the resonating frequency to 1.56 GHz, 2.61 GHz, and 2.93 GHz, with a maximum amplitude of − 13.26 dB, − 18.68 dB, and − 20.18 dB, respectively. The insertion of multiple splits at the edges resulted in a shift in resonance towards higher frequencies. When the splits are eliminated from all edges, the structure shows resonance at 1.62 GHz. The present optimization method has demonstrated the significance of carefully locating a capacitive split at the resonant frequency.

**Table 3:** Computed results of S_21_ and *f*_0_ for multiple capacitive gaps.

Split Gap (SG)	S_21_ (dB)	Frequency (GHz)
**Case 1 (1 SG)**	**− 33.65**	**1.06**
Case 2 (2 SG)	− 13.26	1.56
Case 3 (3 SG)	− 18.68	2.61
Case 4 (4 SG)	− 20.18	2.93
Case 5 (No SG)	− 38.72	1.62

Significant values are in bold.

**Fig. 17:**
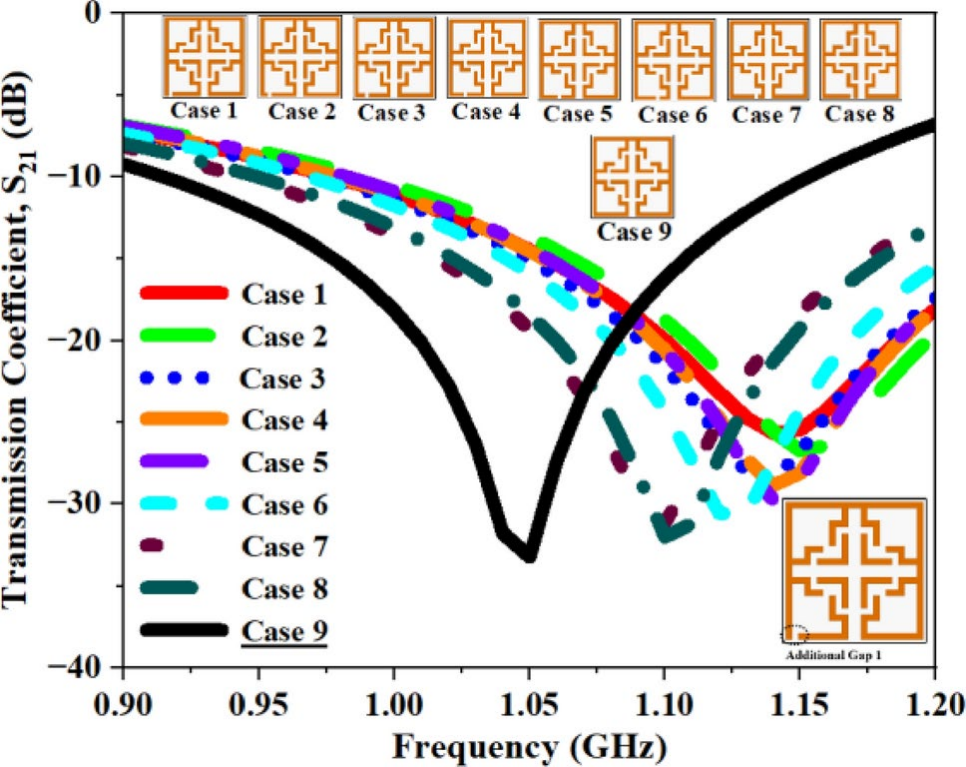
S_21_ plot for one additional gap.

**Fig. 18:**
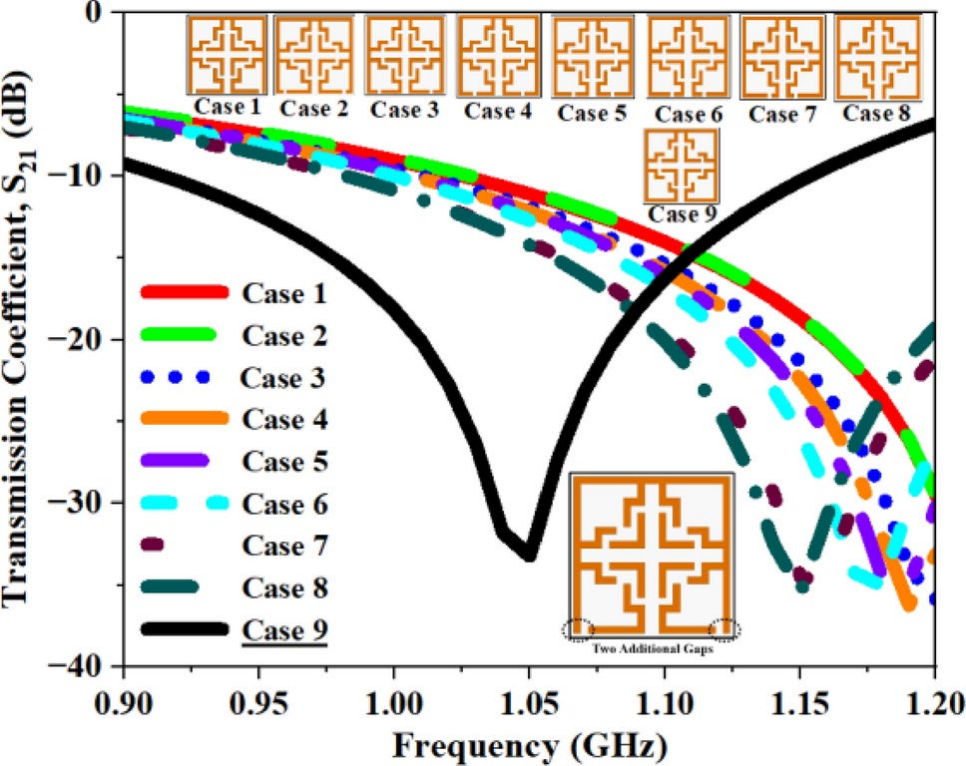
S_21_ plot for two additional gaps.

**Fig. 19:**
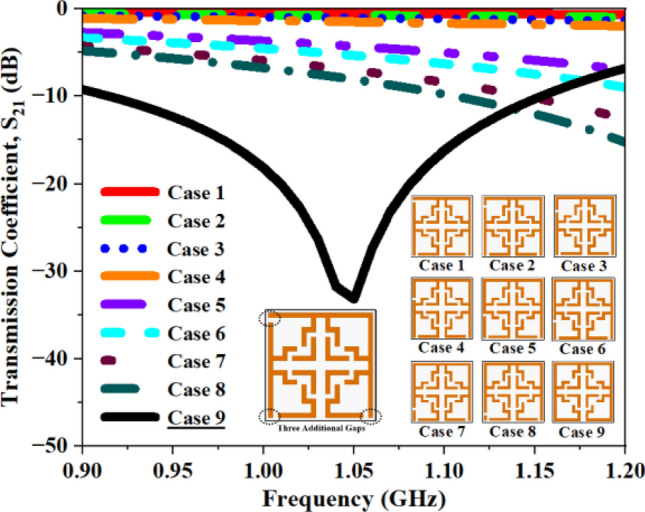
S_21_ plot for three additional gaps.

**Fig. 20:**
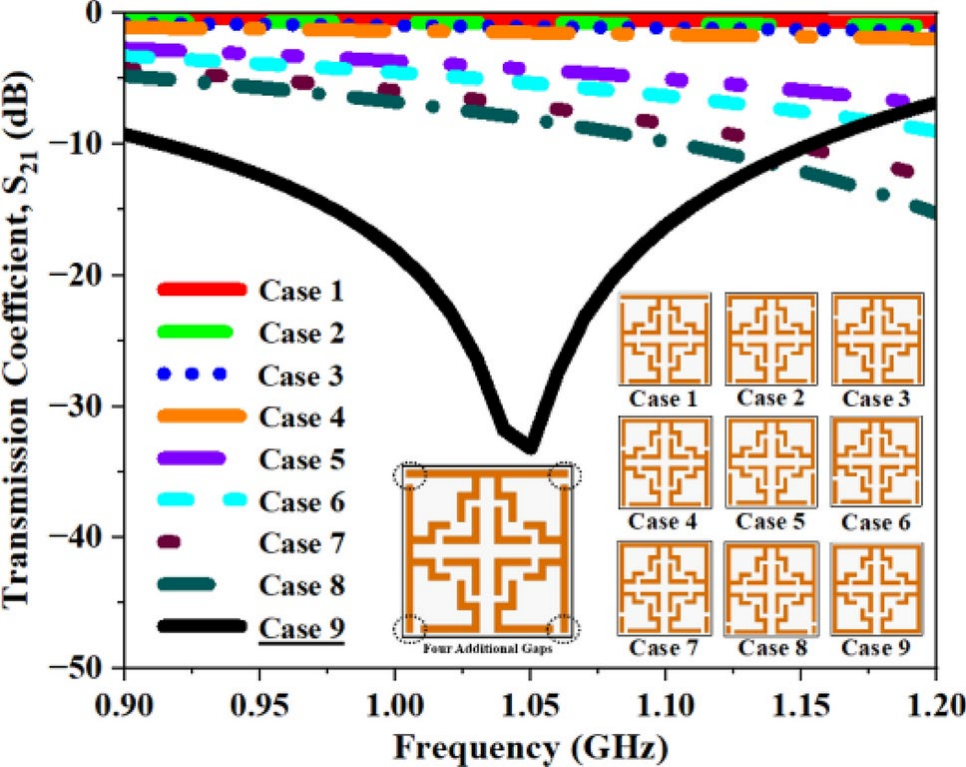
S_21_ plot for four additional gaps.

Alongside the 2 mm CG, supplementary configurations including a single CG (Fig. 17), dual CGs (Fig. 18), three CGs (Fig. 19), and four CGs (Fig. 20) are incorporated to analyze the impact of various gaps on overall performance. The variation in S_21_ and f_0_ values for all the structures are summarized in Tables 4, 5, 6, and 7. The recorded data confirms that the inclusion of CGs will increase the resonant frequency; hence, our design retains only one CG to attain the desired resonant frequency.

**Table 4:** Computed results of S_21_ and *f*_0_ for multiple capacitive gaps.

One AG	S_21_ (dB)	Frequency (GHz)
Case 1	− 25.67	1.14
Case 2	− 26.73	1.15
Case 3	− 28.53	1.14
Case 4	− 28.87	1.13
Case 5	− 29.74	1.14
Case 6	− 30.62	1.13
Case 7	− 31.17	1.11
Case 8	− 31.98	1.10
**Case 9**	**− 33.19**	**1.06**

Significant values are in bold.

**Table 5:** Computed results of S_21_ and *f*_0_ for two AG.

Two AG	S_21_ (dB)	Frequency (GHz)
Case 1	− 35.87	1.22
Case 2	− 35.72	1.21
Case 3	− 35.86	1.20
Case 4	− 36.23	1.19
Case 5	− 35.04	1.18
Case 6	− 35.01	1.17
Case 7	− 34.49	1.15
Case 8	− 35.40	1.14
**Case 9**	**− 33.21**	**1.06**

Significant values are in bold.

**Table 6:** Computed results of S_21_ and *f*_0_ for three AG.

Three AG	S_21_ (dB)	Frequency (GHz)
Case 1	− 27.09	4.09
Case 2	− 35.06	3.51
Case 3	− 33.19	3.01
Case 4	− 34.46	2.63
Case 5	− 33.38	1.48
Case 6	− 34.73	1.42
Case 7	− 35.61	1.34
Case 8	− 35.68	1.31
**Case 9**	**− 33.19**	**1.06**

Significant values are in bold.

**Table 7:** Computed results of S_21_ and *f*_0_ for four AG.

Four AG	S_21_ (dB)	Frequency (GHz)
Case 1	− 27.40	4.11
Case 2	− 31.06	3.53
Case 3	− 33.19	3.02
Case 4	− 34.46	2.63
Case 5	− 33.38	1.48
Case 6	− 34.73	1.42
Case 7	− 35.61	1.34
Case 8	− 35.68	1.31
**Case 9**	**− 33.19**	**1.06**

Significant values are in bold.

#### Analysis of open stubs

**Fig. 21:**
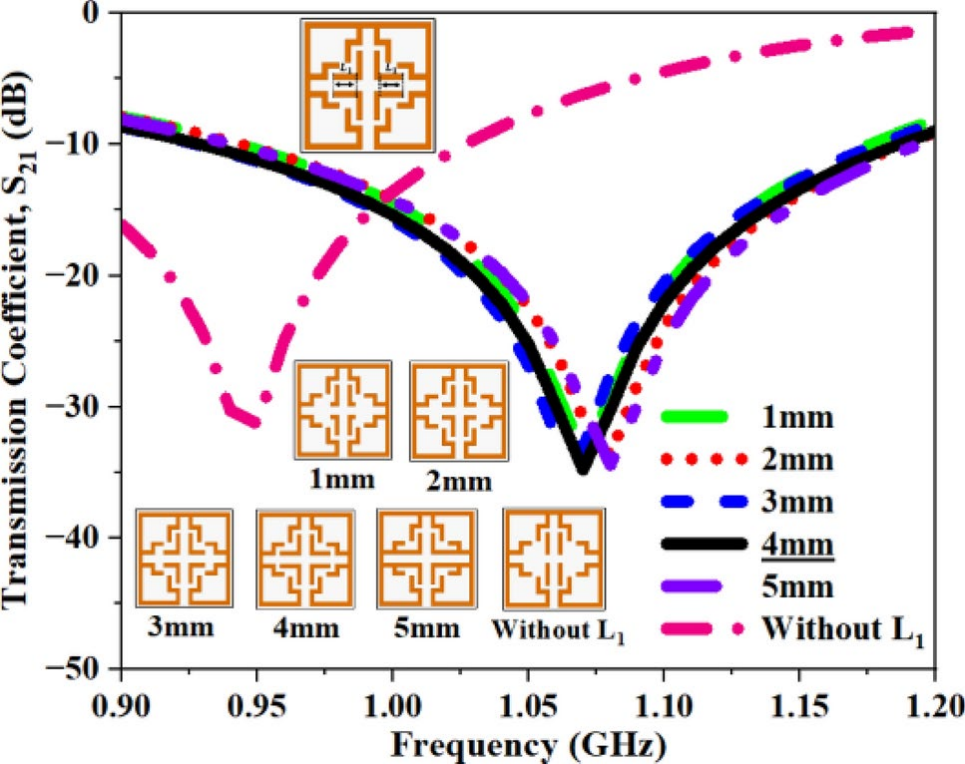
S_21_ plot for open stub L_1_.

This section analyzes the influence of open stub (OS) dimensions and their existence on the output parameters. The horizontal length of the center OS is initially studied by varying its length from 1 to 5 mm, followed by eliminating it to assess its significance in the design, as depicted in Fig. 21. Table 8 summarizes the appropriate S_21_ ~ values for different conditions. The change in horizontal dimension has a minor impact on antenna performance; however, the removal of this stub has resulted in a shift of the resonant frequency to 0.95 GHz, reflecting an 11\% reduction in resonant frequency and emphasizing the significance of the stub. Based on the recorded values (Table 8), the horizontal length is set to 4 mm.

**Table 8:** Computed results of S_21_ and *f*_0_ for open stub L_1_.

Stub length (L_1_	S_21_ (dB)	Frequency (GHz)
1 mm	− 33.53	1.07
2 mm	− 33.73	1.09
3 mm	− 33.19	1.06
**4 mm**	**− 34.88**	**1.06**
5 mm	− 34.53	1.09
Without L_1_	− 31.29	0.95

Significant values are in bold.

**Fig. 22:**
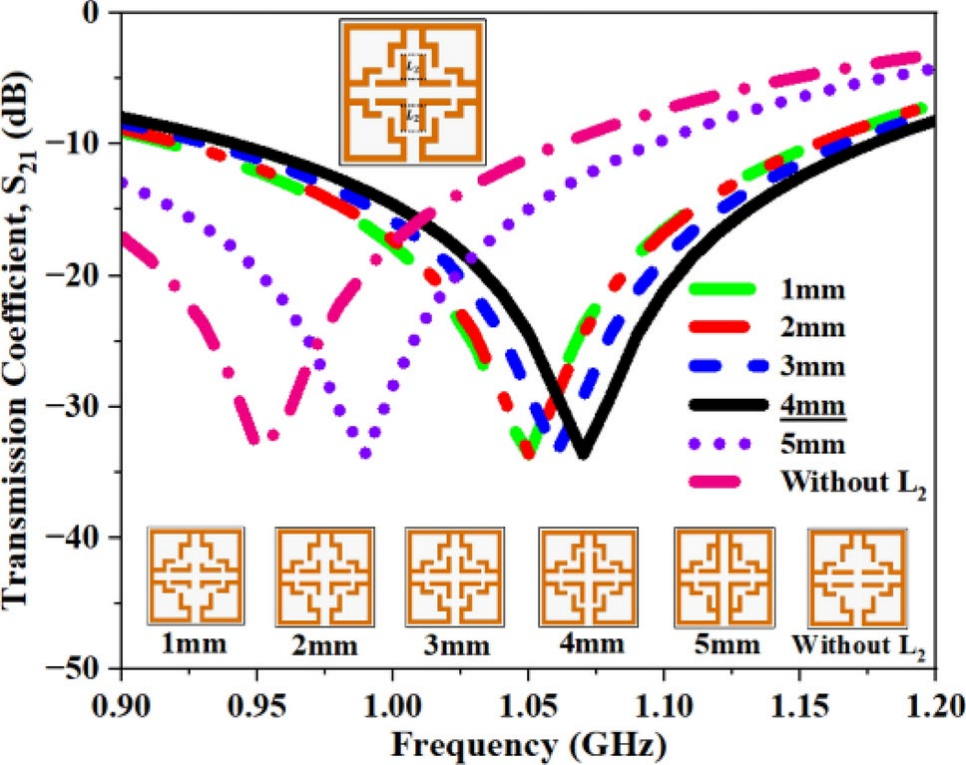
S_21_ plot for open stub L_2_.

Figure 22 illustrates the variation in vertical length and its corresponding S_21_ and f_0_ values are summarized in Table 9.

**Table 9:** Computed results of S_21_ and *f*_0_ for open stub L_2_.

Stub length (L_2_)	S_21_ (dB)	Frequency (GHz)
1 mm	− 33.63	1.05
2 mm	− 33.62	1.05
3 mm	− 33.61	1.05
**4 mm**	**− 33.68**	**1.06**
5 mm	− 33.63	0.99
Without L_2_	− 33.22	0.95

Significant values are in bold.

#### Analysis of closed stubs

**Fig. 23:**
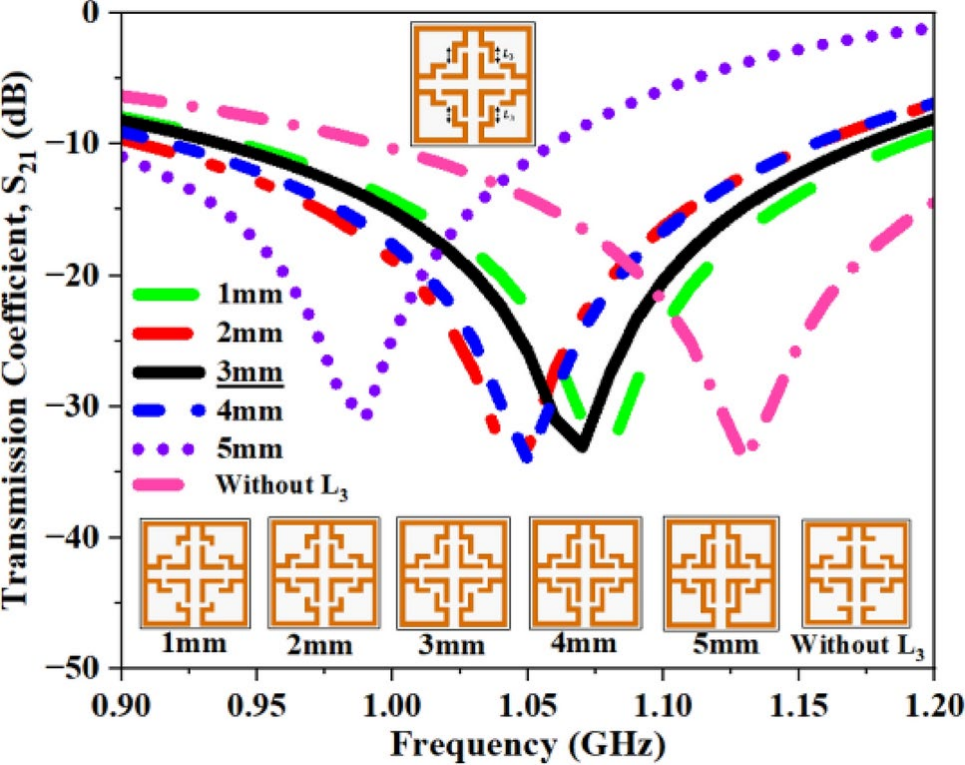
S_21_ plot for closed stub L_3_.

**Fig. 24:**
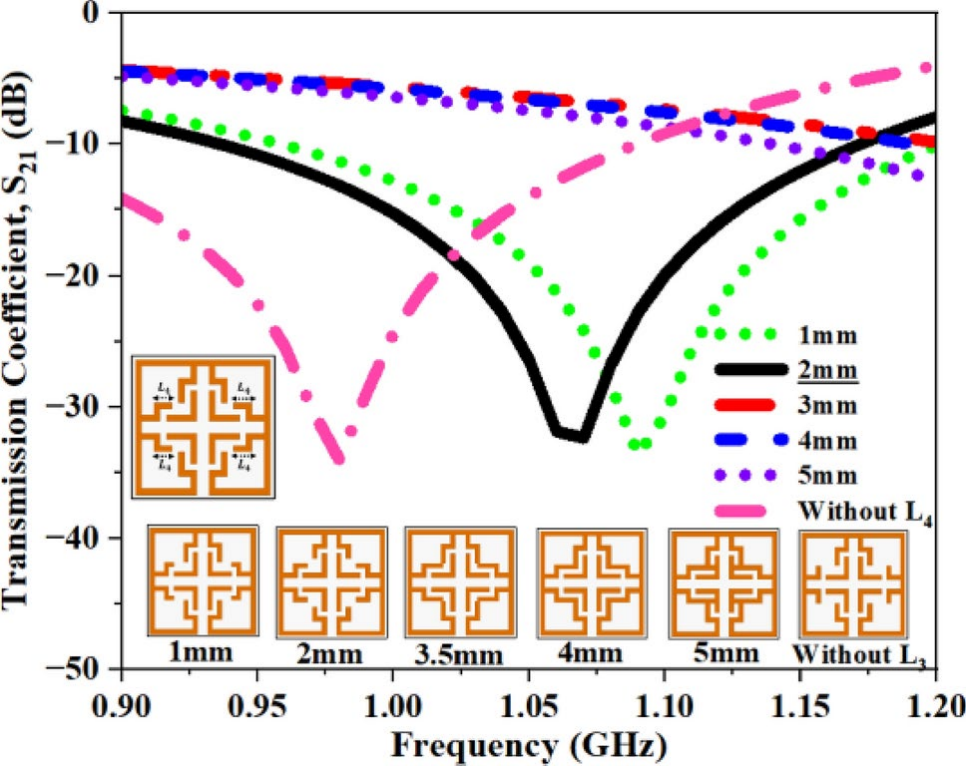
S_21_ plot for closed stub L_4_.

This section examines the impact of closed stub (CS) dimensions and their presence on the output parameters. The horizontal and vertical dimensions of the CSs range from 1 to 5mm, and both stubs were eliminated from the design to assess their significance, as depicted in Figs. 23 and 24.

The S_21_ values for each case are compiled in Tables 10 and 11, and the recorded values clearly demonstrate that alterations in these stubs significantly affect the reflection coefficient. Upon analysis of the recorded values, the lengths of the stubs are set at 3 mm and 2mm, respectively.

**Table 10:** Computed results of S_21_ and *f*_0_ for closed stub L_3_.

Stub length (L_3_)	S_21_ (dB)	Frequency (GHz)
1 mm	− 33.54	1.08
2 mm	− 33.14	1.04
**3 mm**	**− 33.09**	**1.06**
4 mm	− 34.11	1.05
5 mm	− 30.91	0.99
Without L_3_	− 34.19	1.13

Significant values are in bold.

**Table 11:** Computed results of S_21_ and *f*_0_ for closed stub L_4_.

Stub length (L_4_)	S_21_ (dB)	Frequency (GHz)
1 mm	− 33.65	1.09
**2 mm**	**− 32.36**	**1.06**
3 mm	− 35.63	1.43
4 mm	− 35.61	1.41
5 mm	− 34.02	1.33
Without L_4_	− 34.02	0.98

Significant values are in bold.

#### Analysis of relative permittivity of the base material

The relative permittivity (${\upvarepsilon }_{\text{r}}$) of substrate materials substantially influences both resonant frequency and electromagnetic pulses (EMPs).

**Fig. 25:**
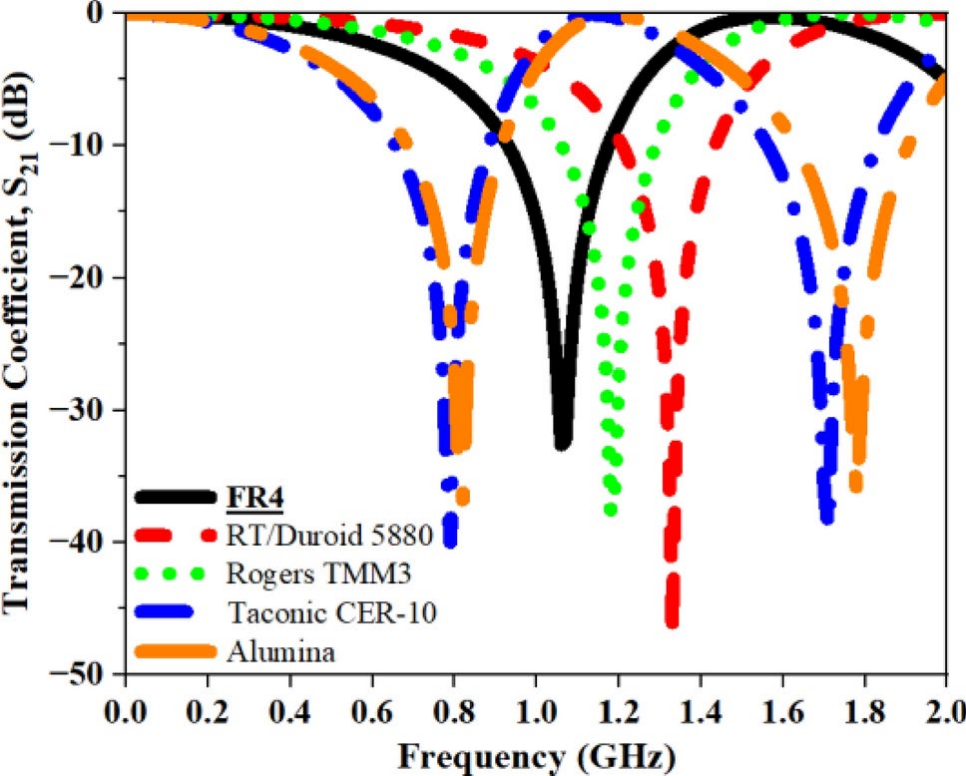
S_21_ plot for different substrate materials.

In this subsection, five distinct base materials, namely FR-4, RT/Duroid 5880, Rogers TMM3, Taconic CER-10, and Alumina, are utilized to examine the effect of base materials on overall performance as illustrated in Fig. 25. The ‘${\upvarepsilon }_{\text{r}}$’ values for these materials are $4.4$, 2.2, 3.27, 10, and 9.2, respectively. Typically, substances with high ‘${\upvarepsilon }_{\text{r}}$’ exhibit a low ${\text{f}}_{0}$ value. The capacitive influence among metallic strips intensifies with an increase in the ‘${\upvarepsilon }_{\text{r}}$’ of the material, leading to a reduction in the ${\text{f}}_{0}$ value. The ${\text{S}}_{21}$ and ${\text{f}}_{0}$ for different base materials are summarized in Table 12. In the current study, we selected the FR-4 substrate due to its cost-effectiveness and availability.

**Table 12:** Computed results of S_21_ and *f*_0_ for different substrate materials.

Substrate Material	S_21_ (dB)	Frequency (GHz)
**FR-4**	**− 32.59**	**1.06**
RT/Duroid 5880	− 46.94	1.33
Rogers TMM3	− 37.75	1.18
Taconic CER-10	− 38.99	1.71
Alumina	− 36.57	1.78

Significant values are in bold.

### Field distribution on the proposed structures

**Fig. 26:**
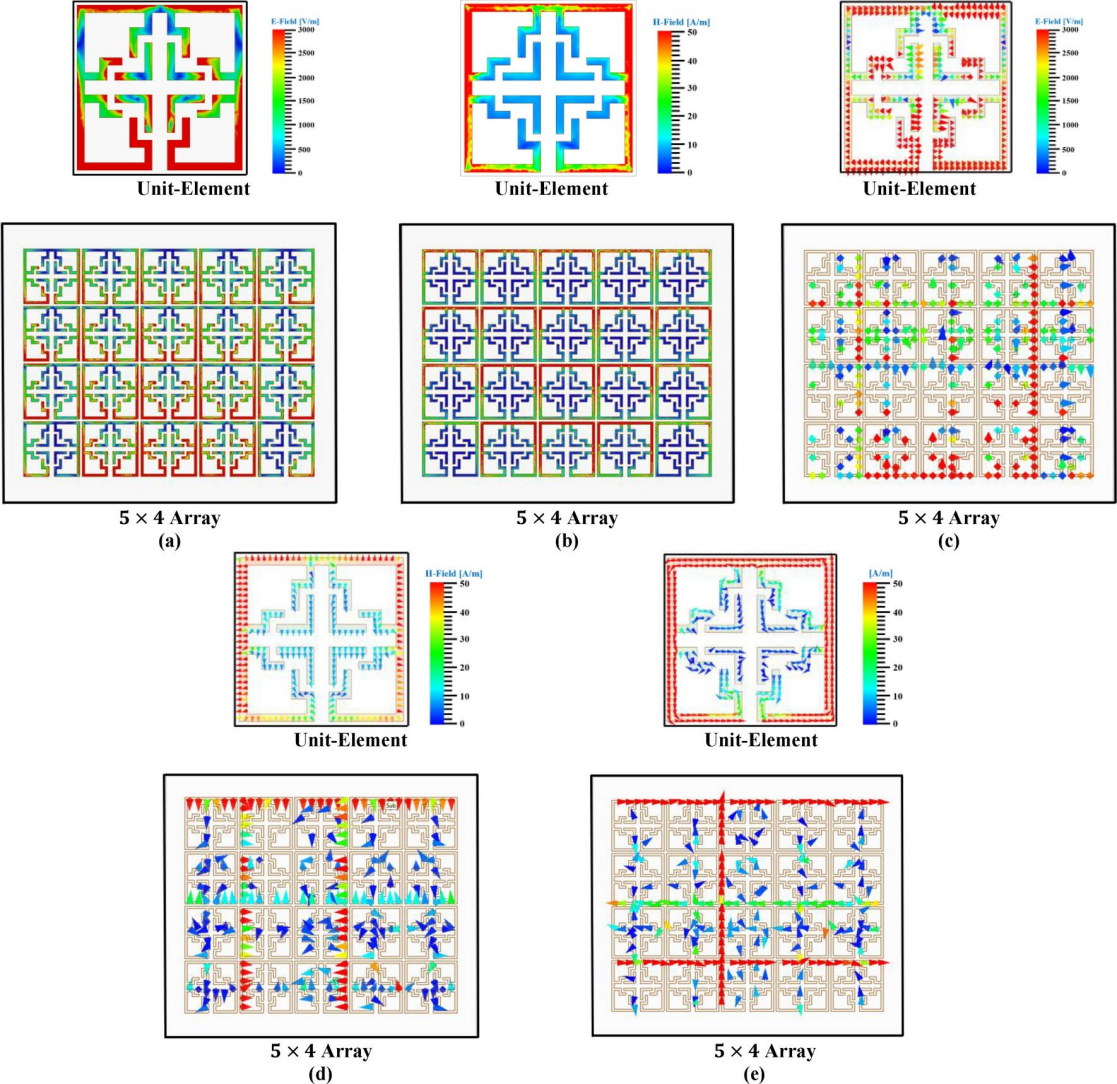
EM-field distribution over unit cell and array structure (**a**) E-field (**b**) H-field (**c**) Vector representation of E-field (**d**) Vector representation of H-field (**e**) Surface current density (J_surf_) in vector form.

In any conducting materials, the surface current signifies the actual electric current induced by an applied time-dependent $\text{EM}$ field. $\text{EM}$ fields are produced by time-dependent electrical impulses in free space, illustrating the interrelation between E and H-fields. The interrelationship between the conducting material, E, and H-field are analyzed by different mathematical modules (conventional law’s and Maxwell’s equations), and the most typical expressions are as follows^29^:

19 $$\nabla \times H = J + \frac{\partial D}{{\partial t}} \quad and\quad \nabla \times E = - \frac{\partial B}{{\partial t}}$$

20 $${\text{D}}\left( {\text{t}} \right) =\upvarepsilon \left( {\text{t}} \right) \times {\text{E}}\left( {\text{t}} \right)\quad {\text{and}}\quad {\text{B}}\left( {\text{t}} \right) =\upmu \left( {\text{t}} \right) \times {\text{H}}\left( {\text{t}} \right)$$

where ‘E’ denotes time-dependent electric intensities, ‘H’ denotes magnetic intensities, ‘D’ denotes E-field density, ‘B’ denotes flux density (H-field), ‘ε’ is electric permittivity, ‘μ’ denotes magnetic permeability, ‘$\text{J}$’ denotes current density in time-dependent electric field, and $\nabla ~\left( {{\text{del\,or\,nabla}}} \right) = \left[ {\partial /\partial x,\partial /\partial y,~\partial /\partial z} \right]$. To evaluate the $\text{EM}$-field of the proposed flower-shaped MTM and array structures, E and H-field distributions, vector representations of these field distributions, and surface current density ‘${\text{J}}_{\text{surf}}$’ plots are considered as seen in Fig. 26.

As illustrated in Fig. 26a, the E-field is intense around the outer ring, particularly in proximity to the capacitive gap, owing to the low impedance effect caused by the gap or split. Additionally, significant fields can be observed around the peripheries of both closed and open stubs due to mutual capacitance. According to Maxwell’s Law, the intensity of the E-field distribution increases as the rate of change of the H-field decreases; hence, in regions with high E-field distribution, the intensity of the H-field will be lower, and vice versa. Figure 26a,b precisely corroborate Maxwell’s Law. The vector field distribution (Fig. 26c,d) provides accurate information regarding field intensity, while the surface current density (Fig. 26e) elucidates the direction of current flow in and around the structure.

## Application of the floral-shape mtm structure

This section thoroughly examines the significance of MTM in enhancing antenna performance. To assess the impact, a radiator operating at 1.06 GHz is initially designed for Flight Navigation Applications (FNAs), and the suggested MTM array structure is employed as a superstrate to gauge the improvements in antenna performance.

### Analysis of MTM loaded FNA antenna

**Fig. 27:**
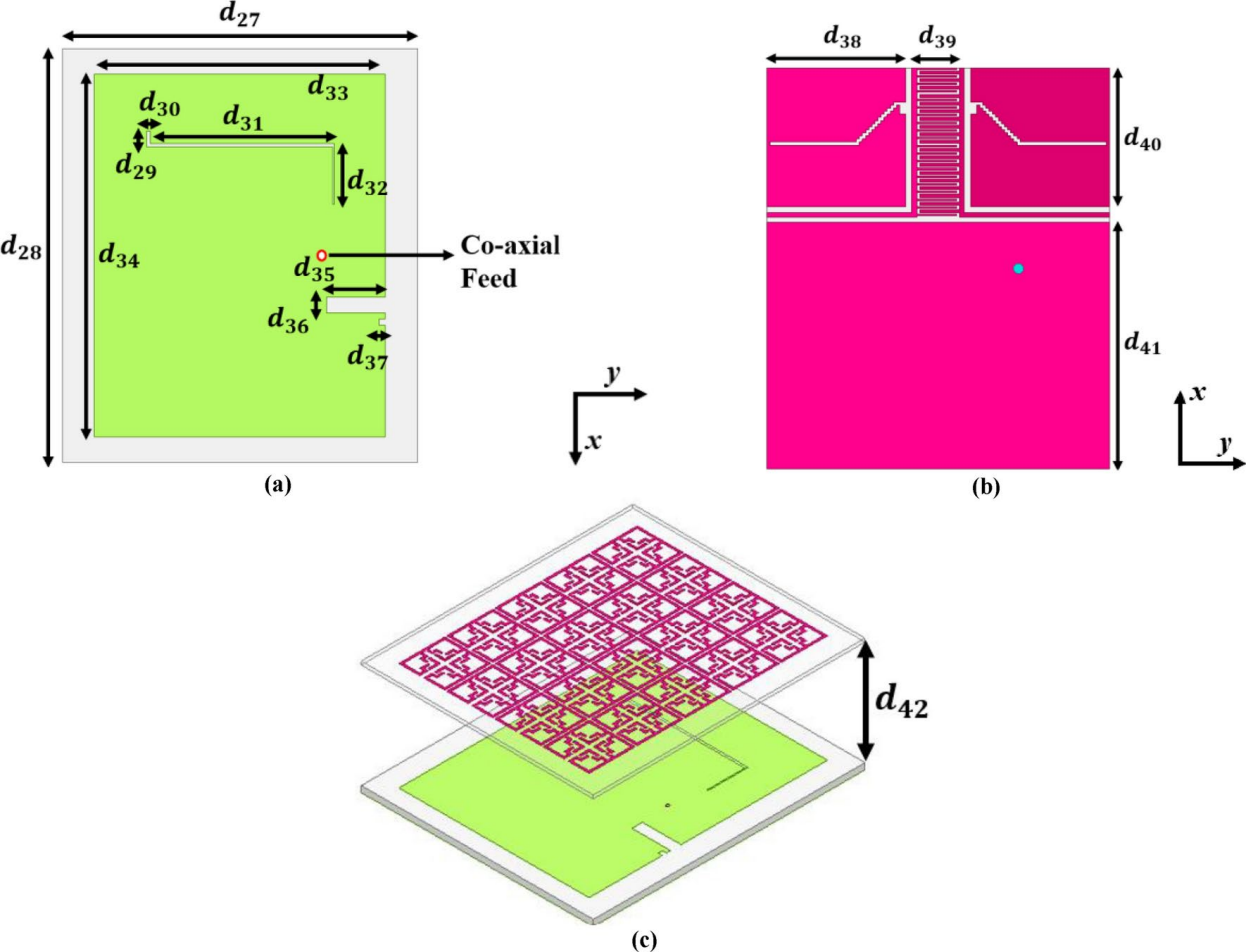
Proposed FNA antenna and MTM loaded FNA antenna (**a**) Top-view (**b**) Ground structure (**c**) Isometric view 5 × 4 MTM array loaded FNA antenna.

Initially, a rectangular radiator is constructed using a $3\text{mm}$ thick RT/Duroid 5880 ($\upvarepsilon _{{\text{r}}} = 2.2$ & $\tan \delta = 0.0009$) base material as illustrated in Fig. 27a–c.

The overall size of a conventional antenna operating at 1.06 GHz is approximated using the Transmission line mathematical model, and the computed dimension is approximately $130\times 111\times 3{\text{mm}}^{3}$^30^.

**Fig. 28:**
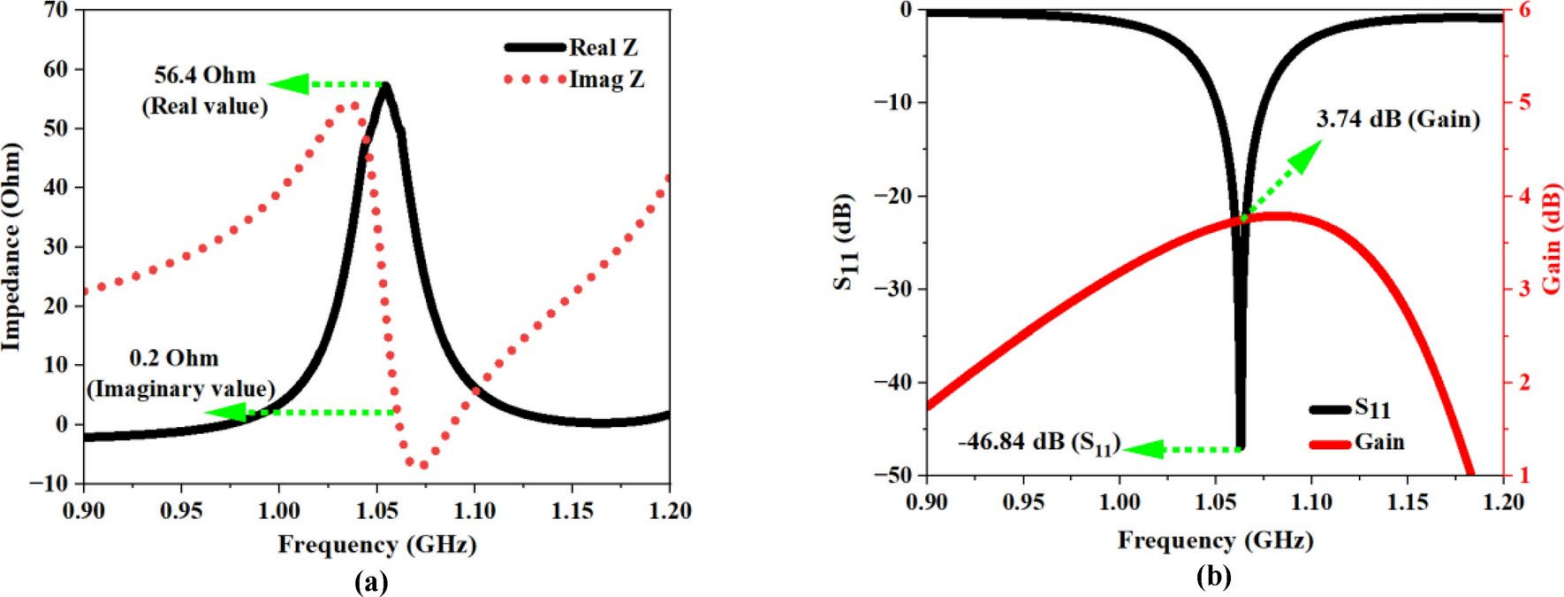
(**a**) Impedance plot of the proposed FNA antenna (**b**) Recorded reflection coefficient (S_11_) and gain data plot.

**Fig. 29:**
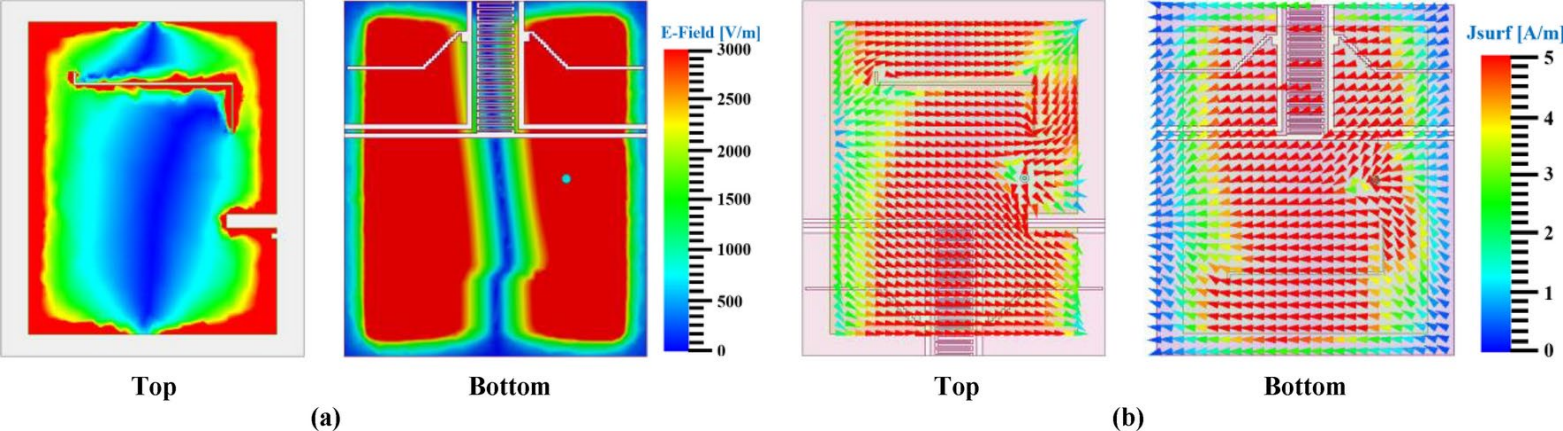
Proposed FNA antenna (**a**) E-field plot (**b**) Surface current density (J_surf_) plot.

The simulation results for the proposed structure are depicted in Fig. 28. The determined impedance of the design radiator is $56.4\Omega$ (Fig. 28a), nearly aligning with the ideal value of $50\Omega$, hence facilitating effective antenna performance within the specified frequency range. Figure 28b illustrates the recorded reflection coefficient and gain data, implying that the design resonates at 1.06 GHz with $-46.84\,\text{ dB}$ reflection coefficient (RC), and a peak gain of $3.74\,\text{dB}$. The E-field dispersion and the surface current density on the proposed FNA antenna is illustrated in Fig. 29a,b.

**Fig. 30:**
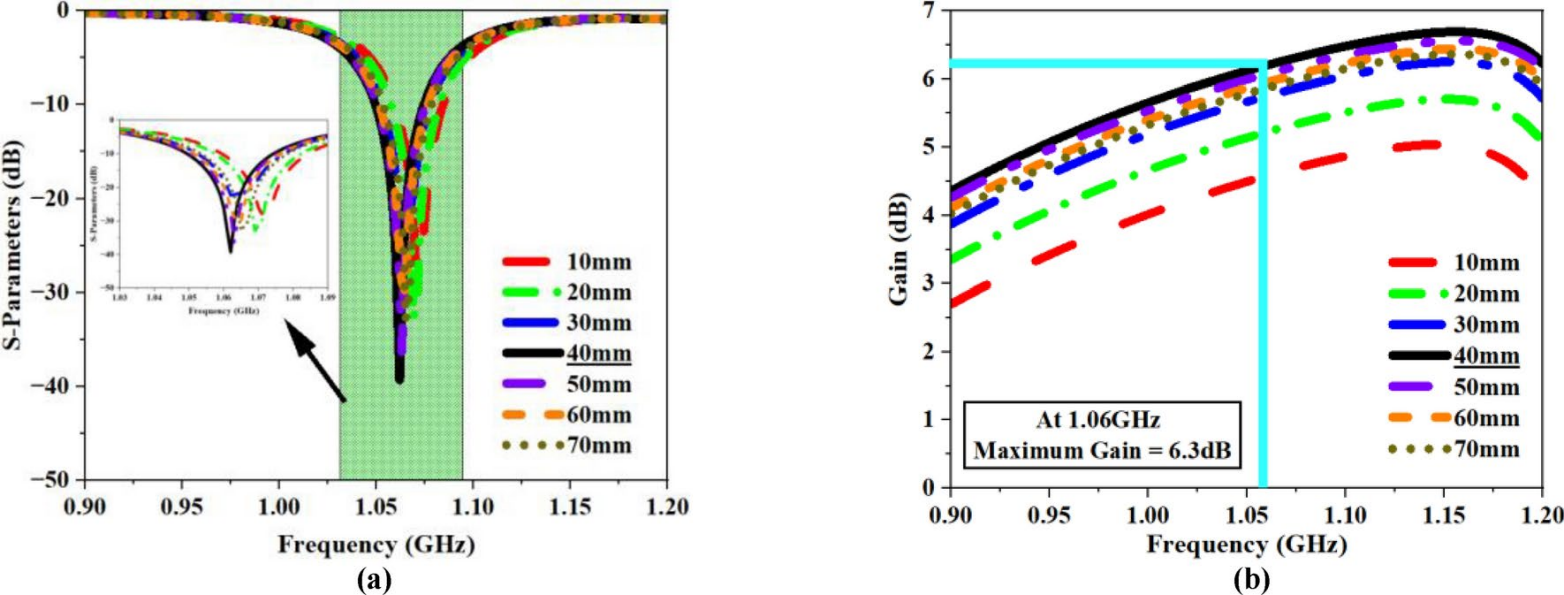
Recorded results of the MTM loaded FNA antenna (**a**) Reflection coefficient plot (**b**) Gain plot.

The proposed MTM array slab is used as a superstrate for the proposed antenna. After placing the MTM slab, the fully enclosed design was simulated numerous times by varying the spacing between two layers from $10\text{mm}$ to $70\text{mm}$. Theoretically, the separation gap between the antenna and the MTM slab can extend to $\lambda /2$. Figure 30 depicts the recorded RC and gain values at different distances.

The distance between two layers does not reduce the RC; in fact, near-consistent RC values can be seen for various separation distances. As illustrated in Fig. 30a, at a distance of $40\text{mm}$, a reflection coefficient of $-40.92\,\text{dB}$ was measured, representing the highest value compared to other separation lengths. Figure 30b clearly demonstrates that the separation distance significantly influences the overall antenna gain.

**Fig. 31:**
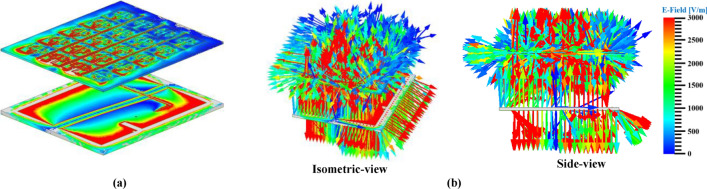
Field analysis of FNA antenna loaded with MTM slab (**a**) E-field plot (**b**) Surface current density (J_surf_) plot.

Theoretically, the propagation of electromagnetic waves across a standard patch or radiator is orthogonal to the $\text{XOY}$ plane. The placement of the MTM slab at an optimal distance from the radiating patch will initially disrupt wave motion; nevertheless, the distinctive material features of the MTM consistently redirect wave propagation along axes parallel to the $\text{XOY}$ plane, thereby augmenting wave intensity in the horizontal plane. The horizontally intensified waves affect the flow of the fundamental beam and its trajectory. Thus, the MTM as a superstrate substantially affects the total antenna gain. The field distribution for the antenna loaded with superstrate layer is illustrated in Fig. 31a,b. In the current configuration, a maximum antenna gain of $6.3\,\text{dB}$ is observed at a separation distance of 40mm between the two layers. Thus, compared to the typical antenna, the superstrate loaded antenna has enhanced the total gain by 2.6dB while retaining the RC value.

**Fig. 32:**
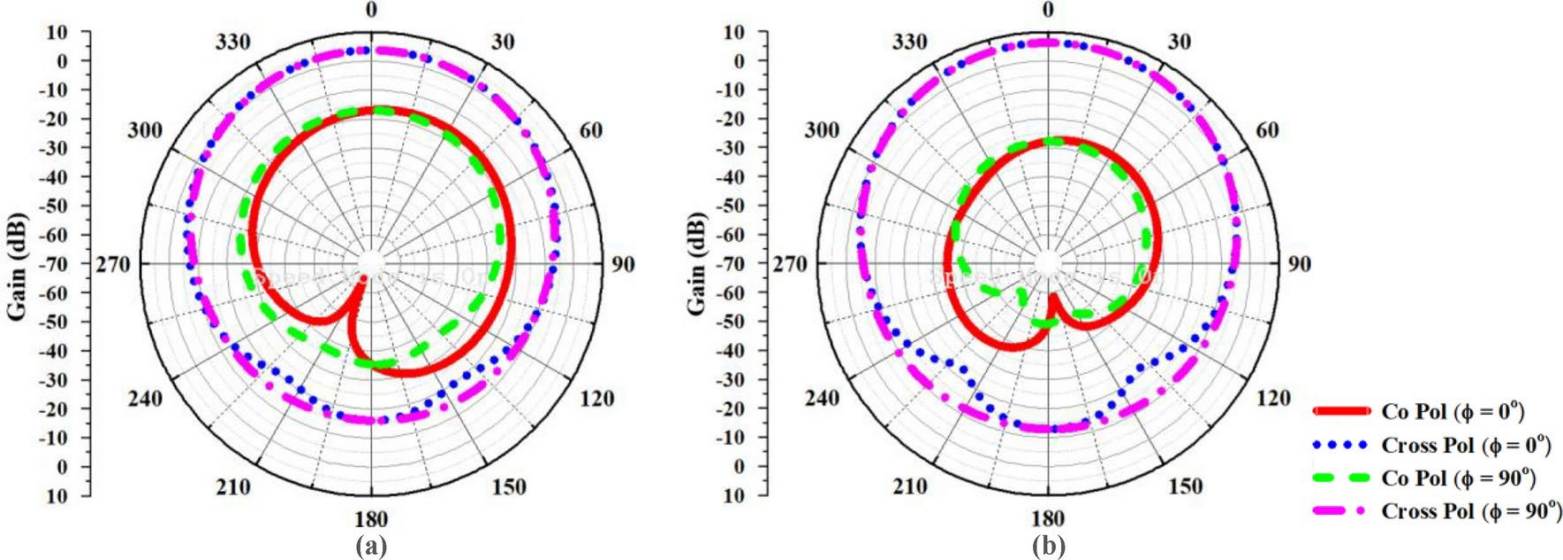
Simulated Co and Cross polarization pattern for XZ and YZ plane (**a**) Proposed FNA antenna (**b**) MTM loaded FNA antenna.

Figure 32a depicts the simulated Co and Cross polarization illustrations for the FNA antenna and the superstrate-loaded FNA antenna. The first plot shows that the radiator primarily radiates EM waves in the intended polarization (co-pol), with negligible losses into the orthogonal polarization (cross-pol). A substantial difference between co- and cross-polarization (below -20 dB) is essential for nearly all applications to ensure effective signal transmission and reception; in this study, a polarization difference below -20 dB is documented for both scenarios.

The second plot as illustrated in Fig. 32b demonstrates a noticeable increase in co-polarization gain relative to the first plot, suggesting enhanced overall performance, apparently owing to the incorporation of the metamaterial superstrate. Minimal cross-polarization signifies excellent polarization purity, demonstrating that the metamaterial superstrate does not substantially disrupt unwanted orthogonal polarization.

### Methodology for gain augmentation using the superstrate concept

In a typical radiator, the conducting or ground layer serves as an obstacle to backward or reverse transmission and modifies the forward patterning according to its dispersion. The maximum attainable gain of a traditional antenna is constrained due to suboptimal luminescence of the reflective layer and the out-of-phase emission from rays reflected from regions with greater distances. Enhanced luminance of the reflective surface can augment antenna gain or directivity; therefore, a partially reflective slab (MTM superstrate layer) is strategically placed near the radiator and adjacent to the reflective elements, elevating the reflection rate and, as a result, illumination. For the greatest possible reflection, the gap between the partial and full reflectors should be adjusted in a way that the rays bounced through a partial slab the medium of air are in phase with their normal trajectory^31,32^.

**Fig. 33:**
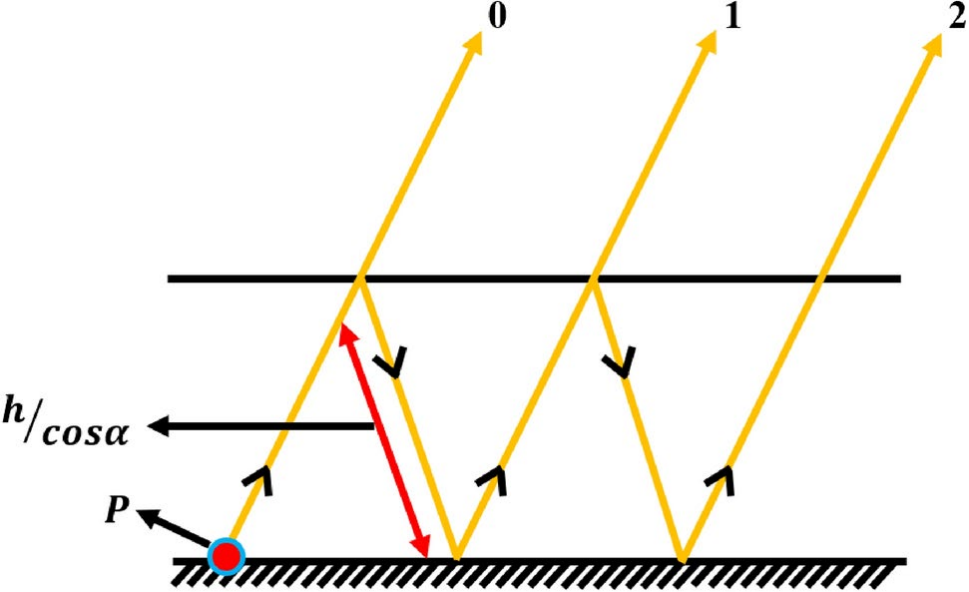
Transmission of waves through two layers.

Presume that the radiator emits a wave characterized by the element sequence $f(\alpha )$ originating from the location P, as depicted in Fig. 33. A MTM slab or partly reflecting surface (PRS) positioned at a constant distance ‘$h$’ above the radiator produces continuous reflections with minimized amplitudes between the layers. Theoretically, the reflection coefficient of the PRS is $p{e}^{j\psi }$, and ideally, the overall transmission loss becomes 0, yielding the magnitude of ray 0 as $\sqrt{1-{p}^{2}}$, the magnitude of ray 1 as $p\sqrt{1-{p}^{2}}$, and the magnitude of ray 2 as ${p}^{2}\sqrt{1-{p}^{2}}$, and so on.

The electric field density in the Fraunhofer zone is determined by the vector summation of the reflected radiation, and for partially and infinitely reflecting layers, the E-field is articulated as^33^

21 $$E = \mathop \sum \limits_{n = 0}^{\infty } f\left( \alpha \right){ }E_{0} p^{n} \sqrt {1 - p^{2} e^{{j\theta_{n} }} }$$

where ${\theta }_{n}$ denotes the phase inclination that characterizes the phase variations of waves as they traverse across two surfaces, as well as the path deviation across two rays. The phase disparity across ray 0 and 1 is expressed as

22 $$\theta_{1} = \frac{2\pi }{\lambda }2h{ }\tan \alpha { }\sin \alpha - \frac{2\pi }{\lambda }\frac{2h}{{\cos \alpha }} - \pi + \varphi$$

Similarly, in between rays 2 and 1 are

23 $$\theta_{2} = \frac{2\pi }{\lambda }4h{ }\tan \alpha { }\sin \alpha - \frac{2\pi }{\lambda }\frac{4h}{{\cos \alpha }} - 2\pi + 2\varphi$$

Thus, for ‘$n$’ wave reflections, the phase variation becomes

24 $$\theta_{n} = n\Phi = n\left[ { - \frac{4\pi }{\lambda }h\cos \alpha - \pi + \varphi } \right]$$

In general, the wave amplitude ($p$) is usually smaller than zero; therefore, the culmination of the RC is

25 $$\mathop \sum \limits_{n = 0}^{\infty } pe^{j\Phi } = \frac{1}{{1 - pe^{j\Phi } }}$$

Incorporating this into Eq. (21), the optimum electric field intensity becomes

26 $$\left| E \right| = \left| {E_{0} } \right|f\left( \alpha \right)\sqrt {\frac{{1 - p^{2} }}{{1 + p^{2} - 2p\cos \Phi }}}$$

Consequently, the power distribution $S$ is defined by,

27 $$S = \frac{{1 - p^{2} }}{{1 + p^{2} - 2p\cos \left( {\varphi - \pi - \frac{4\pi }{\lambda }h\cos \alpha } \right)}}f^{2} \left( \alpha \right)$$

As both amplitude and phase are related to incident angle ‘α’, maximum output at α = 0° is attained when

28 $$\varphi - \pi - \frac{4\pi }{\lambda }h = 0$$

The resonance spacing ‘$h$’ refers to the spatial interval between the patch and the PRS, and is articulated as

29 $$h = \frac{{\varphi_{1 } + \varphi_{2} }}{\pi } \frac{\lambda }{4} + N\frac{\lambda }{2},\quad N = 0,1,2 \ldots$$

here, $\varphi_{1}$ and $\varphi_{2}$ denotes the reflection phases of the ground layer and the MTM layer, and λ signifies its operational wavelength.

The RP of any conducting surface is phi, and since the ground of any radiator serves as a conducting surface, its RP gets rather near to $\uppi$. Therefore, Eq. (29) can be simplified to

30 $$h = \left( {1 + \frac{{\varphi_{2} }}{\pi }} \right) \frac{\lambda }{4}$$

For the proposed application (${f}_{0}$ is 1.06 GHz), the ‘$\lambda$’ is approximately 283 mm, allowing the reflection phase of the radiator to be adjusted between $0$ to $\pi$. Thus, the maximum spacing between the patch and the PRS will be $\lambda /2$ (ranging from 0 to 142 mm).

The fundamental concept concerning how the MTM slab as a superstrate helps to improve antenna capacity can be conceptually analyzed by evaluating the movement of waves across both typical and superstrate loaded antennas, as illustrated in Fig. 31. This analysis facilitates the evaluation of alterations in the transmission direction or concentration of electromagnetic waves due to the integration of the MTM layer at a certain distance from the main radiator.

**Fig. 34:**
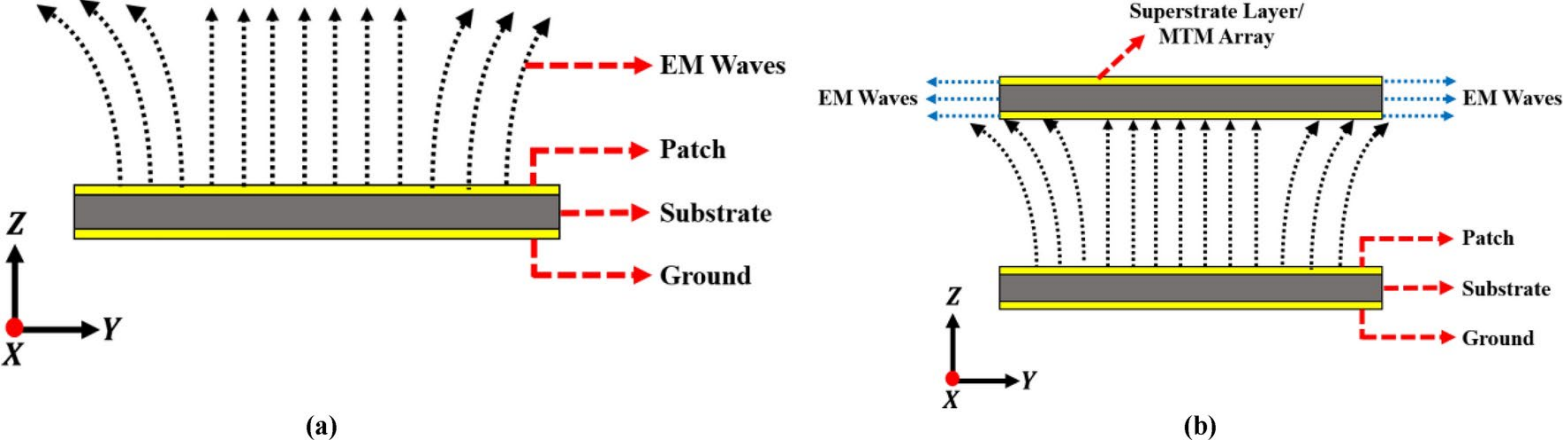
Gain enhancement technique (**a**) Without MTM Superstrate (**b**) With MTM Superstrate.

The role of the MTM superstrate layer on the field dispersion and elevation of antenna gain can be examined using Fig. 34a,b. In the presented FNA antenna (Fig. 27a), the movement of waves is orthogonal to the orientation of the XOY field. The incorporation of MTM superstrate as an intermediary level will predominantly modify wave dispersion; nevertheless, the DNG characteristic of the MTM diverts wave movement in an orientation perpendicular to XOY, leading to intensified wave concentration in the horizontal plane. The augmentation of wave concentration in the horizontal direction affects the trajectory of the primary beam, therefore the substantial concentrated wave on that plane enhances directivity/gain; hence, antennas with superstrate loading substantially enhance overall gain. Figure 31b illustrates the field dispersion and vector model of a simulated superstrate loaded antenna, demonstrating that the MTM layer redirects wave motion and amplifies wave intensity in the horizontal direction, as elucidated by the fundamental principle of gain improvement mechanism (Fig. 34b).

The proposed structure is compared with previously reported papers, and the performance is summarized in the Table 13. To compare, numerous critical metrics, including compact dimensions, achieved gain, and structural originality, among others, are utilized to evaluate the balanced design methodology of the proposed structure. The Table comprises MTM structures functioning within a frequency spectrum of 1.06 GHz to 2.86GH $\text{z}$ (comparatively lower frequencies). The dimensions of the UE are an essential design consideration that influences the efficacy, application, and manufacturing flexibility of the structure. A compact UE facilitates enhanced integration and broader application potential. Unlike existing literature, the proposed structure is remarkably compact, featuring a 13.47 EMR value. Furthermore, using this design as a superstrate layer has a minimal impact on the radiator BW, striking an optimal balance between performance and compactness.

**Table 13:** Comparison of the present work with the reported documents.

References	Year	*f*_0_ (GHz)	UE Dimension	UE Technique	UE Shape	UE Type	Gain Enhancement Technique	Gain ↑ (dB)	*λ*_0_/*a*	Applications
^11^	2022	2.78	10 × 10.5 × 1.6mm^3^ 0.1 × 0.1 × 0.005*λ*^3^	SRR	Coupled circle & square shape (CCS-shape)	ENG	Superstrate	2.45	10.7	MB
^12^	2021	2.86	10 × 10 × 1.5mm^3^ 0.1 × 0.1 × 0.014* λ* ^3^	SRR	Spider web	ENG	Superstrate	2.1	10.48	ATC & WR
^13^	2021	2.40	18 × 16 × 1.5mm^3^ 0.2 × 0.1 × 0.014* λ*^3^	FSS	Spiral	DNG	Superstrate	2		UAV
^34^	2023	2.45	16.5 × 16.5 × 1.6mm^3^ 0.1 × 0.1 × 0.014* λ*^3^	AMC	CCS-shape	DNG	Superstrate	2.76	7.41	IoV
^35^	2022	2.40	12 × 12 × 0.8mm^3^ 0.1 × 0.1 × 0.011* λ*^3^	SRR	Coupled square	DNG	Superstrate	2.5	10.5	PPC
^36^	2022	2.40	22.5 × 22.5 × 1.6mm^3^ 0.18 × 0.18 × 0.013* λ*^3^	SRR	Fractal G-shape	NZRI	Superstrate	2	5.56	WLAN/WiMAX
^37^	2023	1.06	22 × 22 × 1.6mm^3^ 0.078 × 0.078 × 0.005* λ*^3^	SRR	E-shape	DNG	Superstrate	2.71	12.86	ASP
^38^	2024	2.45	21 × 21 × 0.5mm^3^ 0.18 × 0.18 × 0.005* λ*^3^	Dual SRR with Vias	Square Bracket	DNG	Superstrate	3.2	5.84	WLAN
^39^	2024	1.06	22 × 22 × 1.6mm^3^ 0.08 × 0.08 × 0.005* λ*^3^	FSS	Coupled L and U shape	DNG	Superstrate	2.09	12.86	ASP
**PW**	**2024**	**1.06**	**21 × 21 × 1.6 mm**^**3**^ **0.08 × 0.08 × 0.005*λ***^**3**^	**SRR**	**Flower shape**	**DNG**	**Superstrate**	**2.6**	**13.47**	**ASP**

*PW* proposed work, *NZRI* Near Zero Refractive Index, *MB* multi-band, *ATC* air traffic control, *WR* weather radar, *UAV* unmanned aerial vehicle, *IoV* internet of vehicle, *PPC* point-to-point communication, *ASP* aircraft surveillance application.

Significant values are in bold.

MTM structures such as SRR, FSS, and AMC exhibit distinct functionality and are chosen according to the specific needs of the intended application. SRR is a type of MTM configuration that generates negative μ within designated frequency ranges. It comprises concentric metallic loops with tiny gaps, yielding robust resonances at the specified frequency. These strategies will augment the EM response by creating resonances and offer compact structures and a substantial Q-factor for narrowband frequencies applications. The CCS shape^11^, Spider Web^12^, and the coupled square^35^ SRR structures are utilized for different applications due to its exceptional frequency selectivity. The literature, including^36,37^, and the current study, has employed diverse SRR topologies to attain compactness and resonance optimization.

FSS is a periodic pattern that dynamically attenuates specific frequency spectrums while reflecting or allowing others. In other terms, FSS functions as a spatial filter, regulating wave transmission and reflection at designated frequencies. Reference^13^ proposes Spiral FSS for UAV applications, optimizing compactness and broad frequency selectivity, whereas reference^39^ employs Coupled L and U-shaped FSS to get frequency selectivity appropriate for ASP. The AMC structures emulate the characteristics of ideal magnetic conductors by producing in-phase reflections at designated frequencies, comprising periodic metallic regions or patterns supported by a ground plane. These designs will diminish surface wave degradation and augment radiator gain by bouncing EM waves in-phase, while also providing surface impedance match to eliminate destructive interference. In^34^, a CCS-shape AMC is developed for IoV applications, improving surface impedance to enhance antenna performance. Therefore, the selection among SRR, FSS, and AMC is contingent upon application-specific criteria, including dimensions, BW, gain, and operational intricacy.

The parameter ***λ***_**0**_/*a* signifies the compactness in relation to the functional wavelength: Reduced values imply excellent miniaturization, crucial for areas with limited space. This study attains a ***λ***_**0**_/*a* of 13.47, surpassing the majority of documented designs and rendering it appropriate for compact system integration. The compactness of PW makes it an exemplary choice for applications with stringent size limitations, such as aviation systems.

## Conclusion and future scope

In the present work, the floral-shaped double negative MTM design was proposed to improve the performance of the FNA antenna. The antenna resonates at 1.06 GHz, and the overall band allotted for the operation is between 0.9 to 1.18GHz. A new satellite radio (GPS L2) commences at 1.2GHz, the design of any structure must be exceedingly accurate and must not encroach upon the GPS band. The proposed design is systematically analyzed to assess the coverage information and structural characteristics for various incident inclinations across the azimuth region. The material properties (EMPs) of the proposed design are computed using a MATLAB program derived on Fresnel’s methodology. The MTM unit is configured as a 4 × 5 array and serves as a superstrate or parasitic layer positioned above the radiator operating at 1.06GHz, to evaluate its influence on antenna performance. Compared to the unloaded radiator, the integration of MTM into the radiator (loaded) has augmented the overall antenna gain by 2.6dB, while maintaining the reflection coefficient. To verify the simulated outcomes, the proposed structures (unit cell and array) are fabricated, and measurements show that the results are in line with the simulated values. Thus, the designed structure is highly appropriate for Aircraft navigation applications.

A broadband, highly directed radiator is essential for FNA to broadcast and acquire a focused beam from a designated direction, to encompass a broader monitoring area, and enhance airspace efficacy. Therefore, potential future research on the aforementioned applications may employ the suggested structure to augment the total gain offered by the MIMO antennas. Additionally, the engineered MTM cell may serve as a decoupling layer to improve isolation in MIMO antenna systems for FNA applications. The reconfigurability of the radiation pattern and polarization can be attained by employing either PIN or varactor diodes as toggle switches placed over the designated split gaps of the proposed MTM structure.

## Data Availability

The datasets used and/or analysed during the current study available from the corresponding author on reasonable request.
